# Genetic variation in the NBS1, MRE11, RAD50 and BLM genes and susceptibility to non-Hodgkin lymphoma

**DOI:** 10.1186/1471-2350-10-117

**Published:** 2009-11-16

**Authors:** Johanna M Schuetz, Amy C MacArthur, Stephen Leach, Agnes S Lai, Richard P Gallagher, Joseph M Connors, Randy D Gascoyne, John J Spinelli, Angela R Brooks-Wilson

**Affiliations:** 1 Canada's Michael Smith Genome Sciences Centre, BC Cancer Agency, Vancouver, BC, Canada; 2 Cancer Control Research, BC Cancer Agency, Vancouver, BC, Canada; 3 Division of Medical Oncology, BC Cancer Agency, Vancouver, BC, Canada; 4 Pathology, British Columbia Cancer Agency, Vancouver, BC, Canada; 5 Department of Biomedical Physiology and Kinesiology, Simon Fraser University, Burnaby, BC, Canada; 6 School of Population and Public Health, University of British Columbia, Vancouver, BC, Canada

## Abstract

**Background:**

Translocations are hallmarks of non-Hodgkin lymphoma (NHL) genomes. Because lymphoid cell development processes require the creation and repair of double stranded breaks, it is not surprising that disruption of this type of DNA repair can cause cancer. The members of the *MRE11-RAD50-NBS1 *(MRN) complex and *BLM *have central roles in maintenance of DNA integrity. Severe mutations in any of these genes cause genetic disorders, some of which are characterized by increased risk of lymphoma.

**Methods:**

We surveyed the genetic variation in these genes in constitutional DNA of NHL patients by means of gene re-sequencing, then conducted genetic association tests for susceptibility to NHL in a population-based collection of 797 NHL cases and 793 controls.

**Results:**

114 SNPs were discovered in our sequenced samples, 61% of which were novel and not previously reported in dbSNP. Although four variants, two in *RAD50 *and two in *NBS1*, showed association results suggestive of an effect on NHL, they were not significant after correction for multiple tests.

**Conclusion:**

These results suggest an influence of *RAD50 *and *NBS1 *on susceptibility to diffuse large B-cell lymphoma and marginal zone lymphoma. Larger association and functional studies could confirm such a role.

## Background

Non-Hodgkin lymphoma (NHL) is a heterogeneous group of hematological malignancies that in aggregate constitutes the 5^th ^highest cause of cancer mortality in the United States [1] and Canada [2]. NHL subtypes vary in presentation, survival expectation, morbidity and responses to treatment. Chromosomal translocations are so characteristic of NHL that many genes now known to be important in the development of cancer, such as *BCL2 *[3], were originally discovered due to their position at recurrent translocation breakpoints in NHL tumours.

During development and differentiation, the DNA of B- and T-cells is subject to double stranded breaks necessary for the rearrangement of immunoglobulin genes. Genes functioning in double-stranded break repair are involved in successfully controlling and repairing these breaks, thus protecting the genome from molecular events that could lead to cancer. This study examined four genes with key roles in maintaining genome stability: the MRN complex, *MRE11*, *RAD50 *and *NBS1*, and the Bloom syndrome gene (*BLM*). We have previously shown association with NHL of a genetic variant in *H2AX*, which encodes a histone involved in signalling the presence of double stranded breaks [4]. The MRN complex forms foci at sites of double stranded breaks induced by ionizing radiation or immunoglobulin rearrangements during B- and T-cell development, sensing DNA damage and initiating DNA repair [5-7].

The chromosome instability syndromes (reviewed in [8]) form a group of rare autosomal recessive diseases characterized by an increased risk of cancer. This group includes ataxia-telangiectasia (AT, OMIM 208900), Nijmegen breakage syndrome (NBS, OMIM 251260), Bloom syndrome (OMIM 210900) and Fanconi anemia (OMIM 227650). NBS includes an increased risk of lymphoid malignancies [9], particularly B-cell lymphoma [10,11]. Some patients with an NBS-like phenotype have mutations in *RAD50 *[12]. Hypomorphic mutations in *MRE11 *result in an AT-like disorder (AT-LD). NBS and AT-LD share many features, including immunodeficiency and genome instability caused by failure of timely activation of cell cycle checkpoint pathways [13-16].

Mutations in *NBS1 *cause aplastic anemia and acute lymphoblastic leukemia [17,18]. *RAD50 *variants have also been associated with an increased risk of sporadic [12], but not necessarily familial breast cancer [19,20]. MRE11 inactivation has been identified in colorectal cancer cell lines and primary tumours [21], suggesting that inactivation of the MRN complex could be a frequent event in cancers.

Bloom syndrome is also marked by a predisposition to cancer, particularly lymphoma and leukemia in young patients [22]. Although homozygous loss of *Blm *in mice leads to embryonic lethality, heterozygotes show increased risk of neoplasia, with augmented T-cell tumourigenesis [23]. This haploinsufficiency is supported by the increased risk of cancer in *BLM *heterozygotes of Ashkenazi Jewish descent [24], although there is some controversy regarding this finding [25]. This illustrates BLM's role in response to DNA damage [26], particularly during DNA replication stress [27].

While both *Nbs1 *[28] and *Mre11 *[29] null mutants are inviable in vertebrates, the hypermorphic *Rad50*^*S *^mutation causes hematopoietic stem cell failure so that mice that do not die of lymphoma die of bone marrow attrition [30], highlighting the delicate balance the MRN complex exerts on cell survival. This is illustrated by the dosage sensitivity to this mutation and the bidirectional phenotypic rescue in *Rad50*^*S*/*S *^*Atm*^-/- ^mice [31], leading the authors to speculate that while mutations that cause gross chromosomal instability would have a wide array of outcomes, less severe mutations would primarily affect tissues developed from a limited number of precursor stem cells. Since the hematopoietic system is such a system, this reinforces the need to look for variants in genes already known to be associated with severe genetic disorders, with the rationale that varying degrees of mutation severity affect the spectrum of possible effects.

To systematically investigate the role of *NBS1*, *MRE11*, *RAD50 *and *BLM *in susceptibility to NHL, we carried out re-sequencing of these four genes to establish the spectrum of genetic variation in NHL cases, and genotyped 797 NHL cases and 793 controls. Just as total inactivation of a gene and attenuation of its activity lead to different phenotypes in mice, we expected that subtle variation in DNA repair genes could be pertinent to NHL risk in the general population, while complete inactivation of these genes leads to rare and severe syndromes.

## Methods

### Study population

The methodology has been described previously [32,33]. Informed consent was obtained as approved by the joint University of British Columbia/British Columbia Cancer Agency Research Ethics Board. All HIV-negative NHL cases diagnosed in British Columbia from March 2000 to February 2004, residing in the Greater Vancouver Regional District and greater Victoria (Capital Regional District), aged 20 to 79 were invited to participate. Cases were reviewed and coded using the World Health Organization classification by an experienced lymphoma pathologist (RDG). Population controls were identified from the Client Registry of the British Columbia Ministry of Health and were frequency matched to cases by sex, age, and area of residence in a 1:1 ratio. 828 cases and 848 controls completed at least part of a study questionnaire; however, only those subjects with DNA available were used in this study. Table 1 summarizes the characteristics of the 797 cases and 793 controls available for analysis.

**Table 1:** Characteristics of the Study Population.

		Cases (%)	Controls (%)
**Gender**			
	Male	463 (58%)	423 (53%)
	Female	334 (42%)	370 (47%)

**Age group (years)**			
	20-49	150 (19%)	208 (26%)
	50-59	194 (24%)	169 (21%)
	60-69	214 (27%)	206 (26%)
	70+	239 (30%)	210 (26%)

**Ethnicity**			
	Caucasian	625 (78%)	613 (77%)
	Asian	80 (10%)	90 (11%)
	South Asian	29 (4%)	37 (5%)
	Mixed/Other	36 (5%)	34 (4%)
	Unknown/Refused	27 (3%)	19 (2%)

**Pathology**			
**B-cell lymphomas**			
	DLBCL	210 (26%)	-
	FL1	138 (17%)	-
	FL2/FL3	78 (10%)	-
	MZL/MALT	92 (12%)	-
	MCL	47 (6%)	-
	SLL/CLL	43 (5%)	-
	LPL	42 (5%)	-
	MISC BCL	71 (9%)	-
**T-cell lymphomas**			
	MF	40 (5%)	-
	PTCL	29 (4%)	-
	MISC TCL	7 (1%)	-

**Total**		797 (100%)	793 (100%)

DLBCL = Diffuse Large B-Cell Lymphoma, FL = Follicular Lymphoma, FL1 = Follicular Lymphoma grade 1, FL2 = Follicular Lymphoma grade 2, FL3 = Follicular Lymphoma grade 3, MZ/MALT = Marginal Zone lymphoma/Mucosa-Associated Lymphoma Tissue lymphoma, MCL = Mantle Cell lymphoma, SLL = Small Lymphocytic Lymphoma, LPL = Lymphoplasmacytic Lymphoma, Misc. B-cell = Miscellaneous B-cell lymphoma, MF = Mycosis Fungoides, PTCL = Peripheral T-Cell Lymphoma, Misc. T-cell = Miscellaneous T-cell lymphoma.

### DNA extraction and sequencing

Genomic DNA was extracted from whole blood (in 10% of cases from a mouthwash or saliva sample) using the PureGene DNA isolation kit (Gentra Systems) following manufacturer's instructions. DNA was then quantified using PicoGreen (Molecular Probes) in a Victor2 fluorescence plate reader (Perkin-Elmer).

The genomic sequences for all genes were downloaded from the UCSC genome browser [34]. All coding and non-coding exons were sequenced, as well as 1000 base pairs upstream of transcription start. Conserved non-coding sequence regions (CNS regions) were identified using the VISTA genome browser [35]. The six most highly conserved CNS regions with at least 100 base pairs of at least 70% identity with the mouse and rat homolog were also sequenced.

Primers were selected for all amplicons using Primer3 [36]. The -21M13F (TGTAAAACGACGGCCAGT) forward or M13R (CAGGAAACAGCTATGAC) extensions were added to the 5' ends of the forward and reverse PCR primers, respectively, to allow uniform sequencing conditions. PCR and sequencing reactions were carried out as previously described [37]. Primers and conditions used in PCR reactions are listed in Additional file 1. The quality of sequencing reads was assessed using Phred [38,39], potential variants identified by Polyphred version 5 [40] and all sequences assembled with reference sequences using Phrap [41] and viewed in Consed version 12 [42].

Haplotypes of variants with minor allele frequency (MAF) >5% in the sequence data were inferred using PHASE v2.1.1 [43,44]. Four tagSNPs were selected for each gene using TagSNP, version 1.1 [45]. Three additional SNPs of potential functional relevance in NBS1 were also tested.

### Genotyping

TaqMan^® ^was used for all genotyping. Assays were designed using the Assays-by-Design service (Applied Biosystems). Primers and probes used are listed in Additional file 2. 10 ng of each sample was aliquoted in 384-well plates and the DNA dried down at room temperature. TaqMan reactions were carried out in 5 uL volumes as per the manufacturer's protocols. Fluorescence data was obtained in the ABI PRISM 7900 HT, after 10 min at 95°C, followed by 40 cycles of 92°C for 15 s and 60°C for 1 min. The SDS2.2 software (Applied Biosystems) was used to assign genotypes to individual samples.

### Statistical Analyses

Statistical analyses were carried out as described previously [32]. Briefly, all controls were tested for deviation from Hardy-Weinberg equilibrium. Odds ratios (OR) and 95% confidence intervals were estimated using logistic regression. These analyses were conducted using SPSS version 15, with adjustment for sex, age group (categories: 20-49, 50-59, 60-69, 70+), residence (Vancouver or Victoria), and for ethnicity (Caucasian, Asian, South Asian, Mixed, Unknown/Refused) when all cases and all controls were analyzed together. Heterozygotes and rare homozygotes were combined for analysis when the number of rare homozygotes was less than five. Tests were not performed when the sum of the number of heterozygotes and rare homozygotes was less than five for cases or controls. Tests for trend were conducted when there were at least five samples in each genotype category for both cases and controls. Multiple testing correction was carried out by the false discovery rate (FDR) method [46]. Because we tested nineteen markers, the *p*-value of the most significant marker must be below the threshold of 0.0026 to be considered significant. The haplotypes inferred were analyzed as categorical variables and assessed for risk effect using R version 2.1.1 [47]. Haplotypes with frequency <4.5% were combined into a "rare" category.

## Results

### Re-sequencing for variant discovery

We sequenced DNA samples from 87 NHL cases to survey the germline genetic variation in the *NBS1*, *MRE11*, *RAD50 *and *BLM *genes in NHL patients in our population. By using recent methods [48] the number of unseen variants using data from deep sequencing projects (such as ENCODE [49]) can be estimated. Using such methods, the sequencing of 174 chromosomes in our population is expected to have revealed 99.99% of SNPs with a MAF of 1% or more, and 76% of SNPs with a MAF of 0.5% or more. Samples were derived from 74 cases with B-cell NHL and 13 with T-cell NHL (see Additional file 3). The number of amplicons bi-directionally sequenced for each gene is shown in Table 2. In total, 63 amplicons were used. On average, 91.6% of sample-amplicon combinations produced good quality reads in both directions, and 96.6% of sample-amplicon combinations produced good quality reads in at least one direction.

**Table 2:** Gene statistics summary. For *NBS1*, 7 SNPs were genotyped - 4 chosen as tagSNPs and 3 chosen for functional interest.

	Amplicons (bp sequenced)	Missing reads	Missing in both directions	SNPs found by re-sequencing	Coding (non-synonymous)	Singletons	SNPs MAF > 5%	Haplotypes	tagSNPs genotyped	SNPs genotyped
***NBS1***	26 (6042)	6.4%	1.8%	36	8 (6)	14 (39%)	14 (38.9%)	21	4	7

***RAD50***	34 (9031)	6.4%	2.6%	28	8 (4)	17 (61%)	8 (28.6%)	9	4	4

***MRE11***	28 (5999)	12.3%	5.9%	20	2 (1)	11 (55%)	7 (35%)	17	4	4

***BLM***	31 (8537)	25.4%	12.1%	30	11 (6)	13 (43%)	12 (41.4%)	26	4	4

Re-sequencing revealed 114 variants (Additional file [Supplementary-material S4]): 12 small deletions or insertions (10.5%), 73 (64%) transitions and 29 (25.4%) transversions. Twenty-nine variants (25.4%) were in coding regions, with 17 (58.6%) non-synonymous mutations, 4 of which were ranked as "probably" or "possibly damaging" by PolyPhen [50]. Only one of these, BLM_X13_(2603)_C/T, was observed more than once, with a MAF of 5.6%. Fifty-five (48%) variants were "singletons", meaning the minor allele was only observed once in this data set of 87 samples, or 174 chromosomes. Forty-one (36%) variants were "common", with MAF of at least 5%. 59% of variants were previously described in dbSNP (build 128) [51]; their rs numbers are included in Additional file 4. Of the common polymorphisms (MAF ≥5%), 14% were novel.

Overall, sequence variation was found at 34 of 12,352 nucleotides in coding regions (or 8 of 3,805 nucleotides in *RAD50*, 13 of 2,265 nucleotides in *NBS1*, 2 of 2,127 nucleotides in *MRE11*, and 11 of 4,155 nucleotides in *BLM*) and at 95 of 17,257 nucleotides in non-coding regions (or 21 of 5,226 nucleotides in *RAD50*, 32 of 3,777 nucleotides in *NBS1*, 20 of 3,872 nucleotides in *MRE11*, and 22 of 4,382 nucleotides in *BLM*). The *K*_a_/*K*_s _value for these four genes together is 0.6 (or 0.56 for *RAD50*, 0.75 for *NBS1*, 0.50 for *MRE11*, and 0.54 for *BLM*), indicating moderate negative selection.

Linkage Disequilibrium (LD) calculations were performed in sequence data using Haploview v4.0 [52]; singletons were excluded from these calculations. *r*^2 ^values for pairwise combinations of SNPs in each gene are shown in Additional files 5, 6, 7 &8.

### Genotyping

Haplotypes were inferred using the 41 variants that were observed more than once in the sequence data, using PHASE v2.1.1 [43,44]. The number of haplotypes inferred for each gene is indicated in Table 2. Haplotype tagging SNPs (tagSNPs) were selected using TagSNP version 1.1 [45]. Nineteen variants were chosen for genotyping and are indicated in bold in Additional file 4.

The 19 tagSNPs were genotyped in 797 cases and 793 controls, with an average genotype call rate of 97.6%. Their respective MAFs, as calculated using all 1590 samples, are in Additional file 2. The concordance of genotypes (in the 87 samples that were sequenced) between the independent methods of sequencing and TaqMan genotyping was complete; no discrepancies were found. As a quality assurance measure, we also genotyped the 19 SNPs in DNA samples from five three-generation CEPH families (purchased from Coriell Cell Repositories, NJ, USA) and confirmed that the alleles segregated according to Mendelian inheritance.

### NHL association tests

We compared all European ancestry controls against all European ancestry NHL cases, all B-cell NHL, all T-cell NHL and major subtypes individually. One of the variants, MRE11_5UP_(-1456)_C/T, was excluded from analysis due to deviation from Hardy-Weinberg equilibrium in controls. Results for the two most common subtypes - diffuse large B-cell lymphoma (DLBCL) and follicular lymphoma (FL) - and results suggestive of association with Marginal Zone lymphoma/Mucosa-Associated Lymphoid Tissue (MZ/MALT) are shown in Table 3; see Additional file 9 for all results. RAD50_IVS22(+24)_A/G showed a possible association with DLBCL that was strong enough to influence the overall NHL analysis (*p*-trend of 0.022 for DLBCL). Another example is RAD50_IVS7(-38)_C/T in MZL, with an OR of 3.39 (95% CI: 1.48-7.75, p = 0.004).

**Table 3:** Regression analysis in European samples for all SNPs in selected subtypes.

	Controls		DLBCL			FL			MZ/MALT	
SNP	N	N	OR (95% CI)	p value	N	OR (95% CI)	p value	N	OR (95% CI)	p value
**RAD50_IVS4(+19)G/A**										
G/G	384	100	1	*0.896*	107	1	*0.474*	43	1	-
G/A	187	53	1.05 (0.72 - 1.54)	0.799	55	1.08 (0.74 - 1.58)	0.677	19	1.01 (0.57 - 1.80)	0.975
A/A	27	7	0.96 (0.40 - 2.30)	0.935	10	1.31 (0.61 - 2.81)	0.492	3	1.03 (0.29 - 3.61)	0.966
G/A & A/A	214							22	1.01 (0.58 - 1.76)	0.967
**RAD50_IVS7(-38)C/T**										
C/C	568	153	1	-	166	1	-	58	1	-
C/T	34	9	1.01 (0.47 - 2.17)	0.983	6	0.63 (0.26 - 1.53)	0.304	8	3.02 (1.28 - 7.14)	**0.012**
T/T	1	0	0.00 (0.00 -)	1.000	1	3.41 (0.21 - 56.13)	0.391	1	24.77 (1.43 - 427.94)	**0.027**
C/T & T/T	35	9	0.97 (0.45 - 2.08)	0.941	7	0.71 (0.31 - 1.64)		9	3.39 (1.48 - 7.75)	**0.004**
**RAD50_IVS22(+24)A/G**										
A/A	546	137	1	-	150	1	-	59	1	-
A/G	54	25	1.77 (1.05 - 2.96)	**0.031**	24	1.61 (0.96 - 2.71)	0.073	7	1.30 (0.55 - 3.04)	0.550
G/G	1	1	4.54 (0.26 - 78.43)	0.298	0	0.00 (0.00 -)	1.000	0	0.00 (0.00 -)	1.000
A/G & G/G	55	26	1.81 (1.09 - 3.01)	**0.022**	24	1.59 (0.94 - 2.67)	0.081	7	1.29 (0.55 - 3.02)	0.560
**RAD50_IVS22(+62)A/G**										
A/A	601	161	-	-	174	-	-	67	-	-
A/G	2	2	-	-	0	-	-	0	-	-
G/G	0	0	-	-	0	-	-	0	-	-
**NBS1_5(-905)T/C**										
T/T	266	76	1	*0.465*	87	1	*0.847*	28	1	*0.816*
T/C	267	72	0.95 (0.66 - 1.37)	0.769	63	0.75 (0.51 - 1.08)	0.119	43	1.12 (0.65 - 1.93)	0.678
C/C	64	14	0.77 (0.41 - 1.46)	0.423	24	1.20 (0.70 - 2.06)	0.498	7	1.02 (0.42 - 2.48)	0.964
**NBS1_5UTR_(-352)_del(AGTA)**										
AGTA/AGTA	524	138	1	-	137	1	-	60	1	-
AGTA/-	58	16	1.06 (0.59 - 1.92)	0.839	18	1.08 (0.61 - 1.92)	0.785	2	0.30 (0.07 - 1.29)	0.105
-/-	2	0	0.00 (0.00 -)	0.999	3	5.57 (0.90 - 34.52)	0.065	0	0.00 (0.00 -)	0.999
AGTA/- & -/-	60	16	1.03 (0.57 - 1.85)	0.929	21	1.23 (0.71 - 2.11)	0.461	2	-	-
**NBS1_IVS3(+208)G/A**										
G/G	241	66	1	*0.672*	84	1	*0.466*	26	1	*0.818*
G/A	277	76	1.00 (0.68 - 1.45)	0.977	63	0.67 (0.46 - 0.97)	**0.033**	30	0.97 (0.56 - 1.71)	0.935
A/A	78	19	0.86 (0.48 - 1.52)	0.596	27	1.04 (0.62 - 1.72)	0.892	10	1.14 (0.52 - 2.51)	0.744
**NBS1_3UTR(+273)G/A**										
G/G	268	78	1	*0.256*	90	1	*0.509*	26	1	*0.807*
G/A	263	73	0.97 (0.68 - 1.40)	0.885	54	0.65 (0.44 - 0.95)	**0.026**	33	1.28 (0.74 - 2.23)	0.381
A/A	63	11	0.60 (0.24 - 1.19)	0.142	24	1.12 (0.66 - 1.91)	0.669	6	0.89 (0.35 - 2.29)	0.813
**NBS1_X2_(102)_G/A**										
G/G	266	75	1	*0.521*	87	1	*0.935*	28	1	*0.698*
G/A	272	72	0.95 (0.66 - 1.37)	0.782	62	0.72 (0.50 - 1.04)	0.082	33	1.15 (0.67 - 1.97)	0.615
A/A	59	13	0.79 (0.41 - 1.53)	0.486	24	1.31 (0.76 - 2.25)	0.326	7	1.09 (0.45 - 2.67)	0.849
**NBS1_X5_(553)_G/C**										
G/G	255	76	1	*0.306*	84	1	*0.786*	28	1	*0.938*
G/C	270	72	0.90 (0.62 - 1.30)	0.572	62	0.73 (0.50 - 1.06)	0.098	33	1.12 (0.65 - 1.92)	0.688
C/C	57	12	0.70 (0.36 - 1.39)	0.310	22	1.22 (0.70 - 2.13)	0.486	6	0.92 (0.36 - 2.36)	0.857
**NBS1_X13_(2016)_A/G**										
A/A	247	74	1	*0.204*	83	1	*0.634*	27	1	*0.897*
A/G	265	70	0.89 (0.61 - 1.30)	0.549	59	0.69 (0.47 - 1.01)	0.055	33	1.13 (0.65 - 1.95)	0.671
G/G	55	10	0.61 (0.29 - 1.26)	0.180	21	1.20 (0.68 - 2.12)	0.537	6	0.94 (0.36 - 2.43)	0.902
**MRE11_5(-1703)A/G**										
A/A	261	76	1	*0.431*	81	1	*0.725*	25	1	*0.100*
A/G	267	60	0.91 (0.63 - 1.32)	0.629	69	0.84 (0.58 - 1.21)	0.341	29	1.20 (0.68 - 2.13)	0.527
G/G	66	15	0.79 (0.42 - 1.47)	0.457	22	1.03 (0.59 - 1.78)	0.919	13	1.95 (0.93 - 4.10)	0.076
**MRE11_5(-1456)C/T**										
C/C	582	153	1	-	165	1	-	67	-	-
C/T	21	9	1.76 (0.78 - 3.96)	0.173	9	1.38 (0.61 - 3.09)	0.441	1	-	-
T/T	1	1	4.32 (0.26 - 72.48)	0.309	0	0.00 (0.00 -)	0.100	0	-	-
C/T & T/T	22	10	1.87 (0.86 - 4.08)	0.115	9	1.34 (0.60 - 2.99)	0.481			
**MRE11_IVS2(+28)G/A**										
G/G	189	51	1	*0.751*	54	1	*0.967*	17	1	*0.144*
G/A	277	81	1.07 (0.72 - 1.59)	0.753	85	1.10 (0.75 - 1.63)	0.623	32	1.42 (0.75 - 2.66)	0.279
A/A	124	30	0.90 (0.54 - 1.49)	0.675	34	0.99 (0.61 - 1.61)	0.959	18	1.69 (0.83 - 3.46)	0.150
**MRE11_IVS9(-60)A/T**										
T/T	270	82	1	*0.419*	77	1	*0.866*	27	1	*0.625*
T/A	255	61	0.75 (0.51 - 1.09)	0.130	79	1.15 (0.80 - 1.66)	0.453	29	1.19 (0.68 - 2.11)	0.542
A/A	70	20	0.96 (0.55 - 1.68)	0.877	17	0.82 (0.45 - 1.47)	0.498	9	1.14 (0.51 - 2.58)	0.752
**BLM_IVS7(+388)C/T**										
C/C	517	140	1	-	148	1	-	62	1	-
C/T	78	22	1.04 (0.62 - 1.73)	0.891	24	1.03 (0.63 - 1.70)	0.908	6	0.62 (0.26 - 1.49)	0.284
T/T	3	0	0.00 (0.00 -)	0.999	1	0.96 (0.10 - 9.65)	0.970	0	0.00 (0.00 -)	0.999
C/T & T/T	81	22	1.01 (0.60 - 1.68)	0.978	25	1.03 (0.623- 1.68)	0.915	6	0.59 (0.25 - 1.43)	0.244
**BLM_IVS7(+798)ins(T)**										
T/T	528	145	1	-	156	1	-	62	1	-
T/-	69	17	0.89 (0.51 - 1.57)	0.689	17	0.90 (0.51 - 1.58)	0.702	5	0.71 (0.27 - 1.84)	0.474
-/-	4	0	0.00 (0.00 -)	0.999	1	1.09 (0.12 - 10.39)	0.939	0	0.00 (0.00 -)	0.999
T/- & -/-	73	17	0.84 (0.48 - 1.47)	0.534	18	0.90 (0.52 - 1.58)	0.722	5	0.67 (0.26 - 1.75)	0.418
**BLM_IVS12(+7)T/C**										
T/T	316	88	1	*0.944*	88	1	*0.696*	33	1	*0.609*
T/C	243	58	0.85 (0.58 - 1.23)	0.389	68	0.99 (0.69 - 1.43)	0.975	29	1.18 (0.69 - 2.02)	0.546
C/C	45	15	1.23 (0.55 - 2.32)	0.533	16	1.21 (0.65 - 2.26)	0.554	5	1.12 (0.41 - 3.07)	0.823
**BLM_IVS21(-60)_del(GAA)**										
GAA/GAA	237	61	1	*0.968*	74	1	*0.727*	37	1	***0.026***
GAA/-	283	81	1.09 (0.75 - 1.60)	0.640	75	0.87 (0.60 - 1.26)	0.452	21	0.46(0.26 - 0.82)	**0.008**
-/-	76	19	0.94 (0.53 - 1.69)	0.840	24	0.98 (0.58 - 1.68)	0.949	8	0.56 (0.24 - 1.27)	0.162

OR = Odds Ratio, CI = Confidence Interval, DLBCL = Diffuse Large B-Cell Lymphoma, FL = Follicular Lymphoma, MZ/MALT = Marginal Zone lymphoma/Mucosa-Associated Lymphoma Tissue lymphoma.

If less than 5 samples were in a category, the analysis is not valid and marked by "-". Analyses were not done for subtypes that had fewer than 5 heterozygotes and minor homozygotes combined. Analysis is adjusted for adjusted for gender, ethnicity, age, and residence.

p-value for test for trend is shown in italic type.

p-values less than 0.05 are in bold.

All results are in Additional file 9.

Analyses for all NHL were performed separately for the Asian and South Asian cases (see Additional file 10). NBS1_3UTR_(+273)_G/A (rs1063053) gave an OR of 5.3 (95% CI = 1.023 - 27.579, *p *= 0.004) in samples of South Asian ethnicity.

Combined analyses of all samples from all ethnicities were also performed, adjusting for ethnicity in the model (data not shown); some SNPs (usually the same as in the European ancestry only analysis) again showed results suggestive of association but failed to reach *p *< 0.05 upon correction for multiple testing. The ethnic diversity of our study population could mask a real signal and so we focused on the European subpopulation.

The haplotypes inferred from individual SNP genotypes were also tested for association with NHL using R version 2.1.1 (data not shown). No haplotype was more significantly associated with NHL than the individual SNPs forming that haplotype.

## Discussion

*RAD50*, *NBS1*, *MRE11 *and *BLM *were re-sequenced in 87 NHL cases to characterize the variation in these genes in NHL cases in our population. All genes had similar numbers of variants and similar nucleotide diversity, albeit slightly greater for *NBS1 *(Table 2). All four genes showed evidence of negative selection, as indicated by a *K*_a_/*K*_s _value of less than one (0.56 for all four genes combined), which we would expect for genes involved in such a conserved and critical process as DNA repair. The most variable gene, *NBS1*, also showed the lowest conservation.

Two SNPs in *RAD50 *were suggestive of association with specific NHL subtypes (Table 3). RAD50_IVS7(-38)_C/T was suggestive of association with MZ/MALT (*p *= 0.004). The low frequency of this allele (MAF 2.6%), and the low incidence of MZL/MALT (12% of our cases) make it difficult to conclusively implicate this marker in a single study. Interestingly, MZL lymphomas usually develop in tissue subjected to chronic antigenic stimulation, for example gastric MALT lymphoma which arises as a result of chronic *Helicobacter pylori *infection. Such tissue, with persistent and accelerated cell lymphoid cell proliferation, may be uniquely susceptible to neoplastic transformation associated with faulty DNA repair. Our results may serve to highlight specific mechanistic hypotheses for further testing in other association studies, or for *in vitro *functional studies. Mechanisms of tumourigenesis, and the basis for NHL susceptibility, may differ between NHL subtypes. Observations such as ours, if replicable, will help us understand the basis for the diversity of NHL types.

We did not find that variants in *NBS1 *conferred an increased risk of lymphoma, as in most other studies [53-57], although there remain some contradictory positive reports [58-61]. In contrast, non-synonymous mutations in *NBS1 *have been shown to be associated with acute lymphoblastic leukemia in German [17] and Polish [62] children. A study by *Rollinson et al *[63] of haplotypic variation in NHL found no increased risk associated with haplotypes of *NBS1 *and *RAD50*; however, they observed the variant rs601341 in *MRE11 *to have a protective effect on FL and a protective effect of an *MRE11 *haplotype on DLBCL. We did not sequence the part of intron 18 where rs601341 is located and so did not explicitly test this SNP. The difference between our results and those of Rollinson *et al*. could be the result of a SNP-specific effect, and/or the different populations studied.

Although there have been other studies of susceptibility to NHL looking at the genes addressed in this study, most have relied on the genotyping of rare variants discovered in studies of the rare recessive syndromes discussed above. Genotyping was generally done using single-strand conformation polymorphisms [17,53,54,56,58,61,62] or by TaqMan [63]. One study [63] used public databases to collect the information on the SNPs in the regions of interest. However, sequencing of germline DNA of patients with sporadic lymphoma to systematically identify genetic variants had not been previously done. Our systematic characterization of these genes provides valuable information on the variation found in these genes in individuals with NHL. Previous systematic investigations of another double-stranded break repair gene, *ATM*, by our group did not reveal any association between common variants in *ATM *and NHL or its subtypes [32]. In contrast, a common SNP in the promoter region of *H2AX *showed a protective effect on NHL and on FL in particular [4].

Limitations of our study include the histological heterogeneity of NHL, which is composed of many subtypes, many of which are rare. Identification of genetic susceptibility factors that differ between subtypes will be limited by the lack of availability of adequate sample numbers for less common subtypes. The clinical diversity of NHL enabled us to make the strongest conclusions only for DLBCL and FL. Our sample is also ethnically heterogeneous, and so has reduced power to detect genetic factors that are present only in specific ethnic groups. Future replication of results in the context of large international consortia, such as the InterLymph Consortium [64], will help to overcome such limitations.

## Conclusion

While the genes in this study were not significantly associated with NHL independently, it is possible that they could modify NHL risk in combination with other variants. Larger studies would be required to detect such gene-gene interactions. Our observation of possible associations of SNPs in RAD50 with DLBCL and MZ/MALT lymphomas may contribute to the refinement of biological hypotheses for confirmation in larger association studies and functional studies. Mechanisms of tumourigenesis, and the basis for NHL susceptibility, probably differ between NHL subtypes. Specific observations such as these will help us understand the etiological basis for the diversity of NHL types.

## Abbreviations Used

NHL: non-Hodgkin Lymphoma; FL: follicular lymphoma; DLBCL: diffuse large B-cell lymphoma; MRN complex: MRE11-RAD50-NBS1 complex; SNP: single nucleotide polymorphism; NBS: Nijmegen breakage syndrome; AT: ataxia-telangiectasia; AT-LD: ataxia-telangiectasia-like disorder; OR: odd's ratio; CI: confidence interval, CNS: conserved non-coding sequence, PCR: polymerase chain reaction, MZ: marginal zone lymphoma, MALT: mucosa-associated lymphoid tissue

## Competing interests

The authors declare that they have no competing interests.

## Authors' contributions

JMS carried out some experiments, participated in the coordination and interpretation of the analysis, and drafted the manuscript. AM performed the statistical analysis and assisted in interpretation. SL carried out experiments. AL coordinated sample collection and interpreted phenotypes. JC and RDG interpreted the medical relevance and pathology, respectively. RPG and JJS participated in the design of the study and JJS advised on the statistical analysis. ABW participated in the study design, coordinated the experiments and helped to draft the manuscript. All authors read and approved the manuscript.

## Pre-publication history

The pre-publication history for this paper can be accessed here:

http://www.biomedcentral.com/1471-2350/10/117/prepub

## Supplementary Material

Additional file 1 **PCR primers & conditions**. For each exon, the primers and temperatures used for each PCR reaction, as well as the product size. Click here for file

Additional file 2 **Genotyping assays: primers, probes, and minor allele frequencies**. Primers and probes used in genotyping assays, with genotyped minor allele frequencies. Click here for file

Additional file 3 **Samples sequenced**. Composition (gender, age and pathology) of the samples used in the re-sequencing phase. Click here for file

Additional file 4 **SNPs discovered by re-sequencing**. Full list of all SNPs found by re-sequencing and their properties (chromosomal position, dbSNP number if known, flanking sequence, nucleotide/codon/amino acid change, and minor allele frequency). Click here for file

Additional file 5 **Linkage disequilibrium between SNPs in *RAD50***. Correlations between SNPs in *RAD50 *as measured by r^2 ^values. Click here for file

Additional file 6 **Linkage disequilibrium between SNPs in *NBS1***. Correlations between SNPs in *NBS1 *as measured by r^2 ^values. Click here for file

Additional file 7 **Linkage disequilibrium between SNPs in *MRE11***. Correlations between SNPs in *MRE11 *as measured by r^2 ^values. Click here for file

Additional file 8 **Linkage disequilibrium between SNPs in *BLM***. Correlations between SNPs in *BLM *as measured by r^2 ^values. Click here for file

Additional file 9 **Regression analysis in Caucasian samples**. Table containing statistical analysis results in Caucasian samples, for overall NHL and all subtypes examined, with the following sections: Additional file 9a: Regression analysis results for *RAD50 *SNPs in Caucasian samples. Additional file 9b: Regression analysis results for *NBS1 *SNPs in Caucasian samples. Additional file 9c: Regression analysis results for *MRE11 *SNPs in Caucasian samples. Additional file 9d: Regression analysis results for *BLM *SNPs in Caucasian samples. Click here for file

Additional file 10 **Regression analysis for overall NHL in Asians and South-East Asian samples**. Table containing statistical analysis results for overall NHL only. Click here for file
