# Supplementary material for: Genetic variation in the NBS1, MRE11, RAD50 and BLM genes and susceptibility to non-Hodgkin lymphoma
Source: BMC Med Genet. 2009 Nov 16;10:117. doi: 10.1186/1471-2350-10-117 (PMC2788526; doi:10.1186/1471-2350-10-117)
Supplement: Additional file 1 — PCR primers & conditions. For each exon, the primers and temperatures used for each PCR reaction, as well as the product size. [file 1471-2350-10-117-S1.PDF]

Additional File 1 - PCR primers and conditions. M13 sequencing extensions in bold capital font. Coding sequence is shown in capitals, intronic sequence in small font.

Gene	Exon(s)	Annealing Temp.	Product Length	Forward primer with sequencing extension	Reverse primer with sequencing extension
RAD50	P1	58	709	**TGTAAAACGACGGCCAGT**GAGCACTGATCGGAATGGAT	**CAGGAAACAGCTATGAC**GCGCACTCACAGCTTCCT
RAD50	P2	58	733	**TGTAAAACGACGGCCAGT**GGTCTTTCGTCTTTTCCCATT	**CAGGAAACAGCTATGAC**GGAATGTGCGCATGTGTATT
RAD50	CNS1	58	377	**TGTAAAACGACGGCCAGT**CAAACGTTTCTTCAACCAGGA	**CAGGAAACAGCTATGAC**TGCTCATGTCAAAGGCACA
RAD50	CNS2	60	635	**TGTAAAACGACGGCCAGT**CAGAGGCATGCTGAGCAGA	**CAGGAAACAGCTATGAC**TTTGGGCATGGGGTAGAAC
RAD50	CNS3	55	427	**TGTAAAACGACGGCCAGT**CCTTTTGAAAGGAGCATAAAATG	**CAGGAAACAGCTATGAC**AAAGCCCTTAGGTTCTTTGGA
RAD50	CNS4	60	512	**TGTAAAACGACGGCCAGT**TGACTTCCTTTTCCAGAGCAA	**CAGGAAACAGCTATGAC**CCCGGGTGACAGAGAAGTG
RAD50	CNS5	58	529	**TGTAAAACGACGGCCAGT**GAGACCAGCCTAACATGGTGA	**CAGGAAACAGCTATGAC**TTGCTAATTAATTTTCATAACTCAGAA
RAD50	CNS6	60	332	**TGTAAAACGACGGCCAGT**TCCAGGAAGGGGATAAGTGG	**CAGGAAACAGCTATGAC**TGTTCAACTATAGAAGGCTTACTCC
RAD50	1	62	435	**TGTAAAACGACGGCCAGT**gtaccgcacccggaagtc	**CAGGAAACAGCTATGAC**TTAACTGAGGCCGAAGCAAG
RAD50	1	57	431	**TGTAAAACGACGGCCAGT**GTGGGAGGAAAGGCTCCAT	**CAGGAAACAGCTATGAC**tcccattttattctccgaaaga
RAD50	2	56	266	**TGTAAAACGACGGCCAGT**catattttatggtaaacttctgtgg	**CAGGAAACAGCTATGAC**gttttccagtgccaagtttt
RAD50	3	60	330	**TGTAAAACGACGGCCAGT**gccttttctcagaaccaaca	**CAGGAAACAGCTATGAC**gaaaacaaccatcaacttacagacc
RAD50	4	55	332	**TGTAAAACGACGGCCAGT**agatacactgaaggttattttaca	**CAGGAAACAGCTATGAC**aaattgcaaacacagttcaa
RAD50	5	60	423	**TGTAAAACGACGGCCAGT**agtgacagcataatatcccactg	**CAGGAAACAGCTATGAC**ttgatttagccagtccacga
RAD50	6	61	344	**TGTAAAACGACGGCCAGT**ctaaatgcctggacctggag	**CAGGAAACAGCTATGAC**atgtggatggcaaaatgga
RAD50	7	60	404	**TGTAAAACGACGGCCAGT**tgaaggatattgaataaggtttggtt	**CAGGAAACAGCTATGAC**gattggcttttcactcagca
RAD50	8	62	308	**TGTAAAACGACGGCCAGT**CAAGCAGATCGCCATCAAG	**CAGGAAACAGCTATGAC**atgccaaaatggagtccaac
RAD50	9	57	495	**TGTAAAACGACGGCCAGT**tatccctgctgagcaacaca	**CAGGAAACAGCTATGAC**acatgagaataagacaaagttgatgt
RAD50	10	57	407	**TGTAAAACGACGGCCAGT**tgcaaacagtaatatttggaaca	**CAGGAAACAGCTATGAC**gacttatgagtgcaaggtaggc
RAD50	11	54	262	**TGTAAAACGACGGCCAGT**cctatagGCTGACAAAGATGA	**CAGGAAACAGCTATGAC**caattaaacaggtaagcatga
RAD50	12	58	424	**TGTAAAACGACGGCCAGT**tgttggcagaatttgtcttgtt	**CAGGAAACAGCTATGAC**tgaaatccaagccattaggc
RAD50	13	58	466	**TGTAAAACGACGGCCAGT**agaaaaagatacaaccgtattcaga	**CAGGAAACAGCTATGAC**gggcataccagctcagagtc
RAD50	14	60	460	**TGTAAAACGACGGCCAGT**ccctgaaaagaacacaatgtcac	**CAGGAAACAGCTATGAC**caggatggggaattctaaaca
RAD50	15	53	318	**TGTAAAACGACGGCCAGT**ttaaagattttgaataatgcag	**CAGGAAACAGCTATGAC**cgttgtccatgtaacagata
RAD50	16	60	433	**TGTAAAACGACGGCCAGT**gggcccacagaaatagcat	**CAGGAAACAGCTATGAC**gggtgacagaacgagactgt
RAD50	17	61	357	**TGTAAAACGACGGCCAGT**gggtcattgaaaggagcaaa	**CAGGAAACAGCTATGAC**agtgtccgacgtggtgctat
RAD50	18	59	373	**TGTAAAACGACGGCCAGT**tggaaactggaacccaatgt	**CAGGAAACAGCTATGAC**TCTTTGTGTTTCTCGCATTCA
RAD50	19	59	357	**TGTAAAACGACGGCCAGT**TTCAAGATGGGAAAGACGACTA	**CAGGAAACAGCTATGAC**gcctctttccttttttgcattt
RAD50	20	61	357	**TGTAAAACGACGGCCAGT**cacttgctgtcaccagttgc	**CAGGAAACAGCTATGAC**caaaggataccaggctgagg
RAD50	21	54	335	**TGTAAAACGACGGCCAGT**TGAACATCAGAAGTTGGAA	**CAGGAAACAGCTATGAC**aaatgagagaaaagatgactaaa
RAD50	22	54	339	**TGTAAAACGACGGCCAGT**accattgaaaatattgaggaag	**CAGGAAACAGCTATGAC**gtaatgctggcatgatgaga
RAD50	23	61	420	**TGTAAAACGACGGCCAGT**caccagccattgttttcctc	**CAGGAAACAGCTATGAC**caaagagcttcccaccattc
RAD50	24	60	340	**TGTAAAACGACGGCCAGT**cctgtgacgtttcccacttt	**CAGGAAACAGCTATGAC**cacaacctgtccccaaatgt
RAD50	25	60	517	**TGTAAAACGACGGCCAGT**caaggtttgcggtgactttt	**CAGGAAACAGCTATGAC**GGGATGAAGAGCAGCAAATC
NBS1	P1	62	504	**TGTAAAACGACGGCCAGT**ggtgaaatccttatcttcatcatt	**CAGGAAACAGCTATGAC**GCAGCAGTTTCCACATCG
NBS1	P2	62	504	**TGTAAAACGACGGCCAGT**ctgggcctaaacaagccttt	**CAGGAAACAGCTATGAC**ttaaatgcattaaaatacttaccagtt
NBS1	P3	65+	505	**TGTAAAACGACGGCCAGT**ttgtgcttaggagtttgcatc	**CAGGAAACAGCTATGAC**gtctacatcctgcgcactact
NBS1	P3b	65+	505	**TGTAAAACGACGGCCAGT**gacagggtcttgctctatcg	**CAGGAAACAGCTATGAC**agctggttcaagctctcctc
NBS1	CNS1	60	330	**TGTAAAACGACGGCCAGT**CGCTGGTGGTAATAAGAAAGATG	**CAGGAAACAGCTATGAC**TGCTTTGGTGCAGTCTGTTT
NBS1	CNS2	53	335	**TGTAAAACGACGGCCAGT**TGGTGAGTGCATTTTGTACATC	**CAGGAAACAGCTATGAC**GTTGCAGAGATGGCAATTTT
NBS1	CNS3	54	335	**TGTAAAACGACGGCCAGT**TTAAGCTTACCCACTATACACAGATTT	**CAGGAAACAGCTATGAC**TTGAAGACTGGCAGTTAATCTCTA
NBS1	CNS4	54	335	**TGTAAAACGACGGCCAGT**CTTTAATGCTTGTTTCAGGATGT	**CAGGAAACAGCTATGAC**TTCTGGACTATGTGGCTTGC

Gene	Exon(s)	Annealing Temp.	Product Length	Forward primer with sequencing extension	Reverse primer with sequencing extension
NBS1	CNS5	58	283	**TGTAAAACGACGGCCAGT**GCCCCAGCGAGTAAGCTATT	**CAGGAAACAGCTATGAC**GGCCCTGAGGATCACAGTAA
NBS1	CNS6	58	433	**TGTAAAACGACGGCCAGT**GCAAATGTACATGGATGACTGG	**CAGGAAACAGCTATGAC**TGGCCTCAATATGAGTGTAGCA
NBS1	1	62	389	**TGTAAAACGACGGCCAGT**actcccgcctcatccaag	**CAGGAAACAGCTATGAC**cttgccatacagcgtactcg
NBS1	2	58	433	**TGTAAAACGACGGCCAGT**ctaacatttgaataacaaaggtgttt	**CAGGAAACAGCTATGAC**caaatcctgtgaactctctctca
NBS1	3	56	443	**TGTAAAACGACGGCCAGT**gccacctgcctattgtcttt	**CAGGAAACAGCTATGAC**ttcctttaggatttggctgaaa
NBS1	4	59	433	**TGTAAAACGACGGCCAGT**aaaaattgccatctctgcaa	**CAGGAAACAGCTATGAC**aaaaatctgtgtatagtgggtaagc
NBS1	5	58	388	**TGTAAAACGACGGCCAGT**gagagatgaaagggaaaaca	**CAGGAAACAGCTATGAC**ccgaactataacacagcaact
NBS1	6	57	433	**TGTAAAACGACGGCCAGT**tggaaattatgccttttgagtg	**CAGGAAACAGCTATGAC**caaaatcccaaaatgaaatacg
NBS1	7	56	445	**TGTAAAACGACGGCCAGT**tgtttcccaaatcaaattcttatg	**CAGGAAACAGCTATGAC**ttgttaggtgaaaagcaacaaaa
NBS1	8	61	411	**TGTAAAACGACGGCCAGT**ccataaaattatttgggaggaaaa	**CAGGAAACAGCTATGAC**tggtgaatatggtcaccccta
NBS1	9	57	445	**TGTAAAACGACGGCCAGT**tcccctcagcatggtatagtc	**CAGGAAACAGCTATGAC**cctggtatcccattcttcca
NBS1	10	56	496	**TGTAAAACGACGGCCAGT**tttaacgatctttgtttctctatt	**CAGGAAACAGCTATGAC**agcagcagaagcatacttaatc
NBS1	11a	60	507	**TGTAAAACGACGGCCAGT**tgtgaactaaatggagggagtg	**CAGGAAACAGCTATGAC**CACATCATCCATTTCCCTTTTT
NBS1	11b	61	504	**TGTAAAACGACGGCCAGT**GCCTGTGGACACAAACTCAG	**CAGGAAACAGCTATGAC**caaaatcgaaagtacctgttagcat
NBS1	12	57	384	**TGTAAAACGACGGCCAGT**ttcaaaaggccaagaagtga	**CAGGAAACAGCTATGAC**aatccatttcaaggcacaatc
NBS1	13	59	452	**TGTAAAACGACGGCCAGT**gattcccaaatgacaagtga	**CAGGAAACAGCTATGAC**tgcttttatctttgtttagcatca
NBS1	14	59	451	**TGTAAAACGACGGCCAGT**ggcacttatgcatgatttacca	**CAGGAAACAGCTATGAC**tgcttgaaggccaccataat
NBS1	15	61	443	**TGTAAAACGACGGCCAGT**cctccaggatgtggaaatct	**CAGGAAACAGCTATGAC**aaacccaattgagaggtgctt
NBS1	16	57	466	**TGTAAAACGACGGCCAGT**ctatttgccaaagtgtgactaca	**CAGGAAACAGCTATGAC**GGGTGACTTTAGTCTTTACCTTACG
MRE11	P1	58	732	**TGTAAAACGACGGCCAGT**GGACCCTTCAATGGAACTGA	**CAGGAAACAGCTATGAC**GCAGGATCCGTGAAAAGAAA
MRE11	P2	60	698	**TGTAAAACGACGGCCAGT**CCTGAACCTTGCAACACAAA	**CAGGAAACAGCTATGAC**GAGCTGGATCCCCTCTCC
MRE11	CNS1	55	428	**TGTAAAACGACGGCCAGT**CGATTTTCCTGGAAGAGTACGA	**CAGGAAACAGCTATGAC**ATTTTAAATGTGTTTTCATCATCACT
MRE11	CNS2	55	378	**TGTAAAACGACGGCCAGT**GATGAAGGTCACCATCTCACAA	**CAGGAAACAGCTATGAC**TCCTTTTCTGTGAAGTATGTCCA
MRE11	CNS3	58	461	**TGTAAAACGACGGCCAGT**TGGAAGTAAGACTAGAGATCCTACAA	**CAGGAAACAGCTATGAC**GCACAAAGTGCATCTGCCTA
MRE11	CNS4	58	324	**TGTAAAACGACGGCCAGT**CATATGCTACCATCATAGTCCTGTT	**CAGGAAACAGCTATGAC**GGAGAGAAATGAACACCGAGTC
MRE11	CNS5	58	335	**TGTAAAACGACGGCCAGT**GCTGCTACTATGTTTGCTACCC	**CAGGAAACAGCTATGAC**TGGAAGGCAAAAGATGAGTG
MRE11	CNS6	58	284	**TGTAAAACGACGGCCAGT**GACATTTTTCCTACCACTTCAAAGA	**CAGGAAACAGCTATGAC**ACAGGCTATTTCAATTTTCACTG
MRE11	2	58	213	**TGTAAAACGACGGCCAGT**TTTGGAATCGAGTGCATTTTC	**CAGGAAACAGCTATGAC**tgatcacagttgacgagcttt
MRE11	3	58	351	**TGTAAAACGACGGCCAGT**ttgtctcaatttgtttgaatatcct	**CAGGAAACAGCTATGAC**aggcaaggtaagcacctgag
MRE11	4	52	334	**TGTAAAACGACGGCCAGT**tctctttcatctgtagtttacaaaaa	**CAGGAAACAGCTATGAC**cgtgtcttatacagcaaatacca
MRE11	5	53	265	**TGTAAAACGACGGCCAGT**tttggaggagaatcttaggg	**CAGGAAACAGCTATGAC**catgctttccacagacaaac
MRE11	6	53	326	**TGTAAAACGACGGCCAGT**tgcttttaacaggtgatacga	**CAGGAAACAGCTATGAC**tctccaaatttctcaattgttt
MRE11	7	56	300	**TGTAAAACGACGGCCAGT**tgagaaggacataatttagaagca	**CAGGAAACAGCTATGAC**tcagaaacagatttgggaagc
MRE11	8	58	433	**TGTAAAACGACGGCCAGT**tcctgtgcacactttctggt	**CAGGAAACAGCTATGAC**ggccttaaacctatgagatgatt
MRE11	9	62	366	**TGTAAAACGACGGCCAGT**tgcacatcaccctgagga	**CAGGAAACAGCTATGAC**tcaacataaaggcttccctct
MRE11	10	56	271	**TGTAAAACGACGGCCAGT**gctgctattcagccaagttaaa	**CAGGAAACAGCTATGAC**tcctcactacttttcaaagaaacc
MRE11	11	59	326	**TGTAAAACGACGGCCAGT**caagcataaacactgtgaatactgaa	**CAGGAAACAGCTATGAC**tttaataaagattccttcacaaatcc
MRE11	12	58	331	**TGTAAAACGACGGCCAGT**ccttctccactgacaacttgc	**CAGGAAACAGCTATGAC**gaaacattttgaggattcattttgt
MRE11	13	56	397	**TGTAAAACGACGGCCAGT**tttggtaattttccttgctca	**CAGGAAACAGCTATGAC**aaacaaatcagagaggttaaatagtga
MRE11	14	58	278	**TGTAAAACGACGGCCAGT**ttcttaattgtagccccttgtt	**CAGGAAACAGCTATGAC**tcccctagacctatggactga
MRE11	15	60	311	**TGTAAAACGACGGCCAGT**tgattagttcatgtcaatgcctaaat	**CAGGAAACAGCTATGAC**AATTCTGCCCTCTTCCACCT
MRE11	15	60	319	**TGTAAAACGACGGCCAGT**AGGGCCAGAGCACTCAGAT	**CAGGAAACAGCTATGAC**gacaagatctaattctgtattttccac
MRE11	16	57	263	**TGTAAAACGACGGCCAGT**ttagtgttgtatgaaataaaccttagc	**CAGGAAACAGCTATGAC**aagggctaccaatggtgatt
MRE11	17	57	264	**TGTAAAACGACGGCCAGT**cagttttgcagataggactcagaa	**CAGGAAACAGCTATGAC**ttgacaattcataatgcagaaaac

Gene	Exon(s)	Annealing Temp.	Product Length	Forward primer with sequencing extension	Reverse primer with sequencing extension
MRE11	18	53	261	**TGTAAAACGACGGCCAGT**aatcgtgtaattcatttcttca	**CAGGAAACAGCTATGAC**tgtgtaactttggaattctgg
MRE11	19	61	257	**TGTAAAACGACGGCCAGT**gccagaaaggcacacttgat	**CAGGAAACAGCTATGAC**tcagcaactagctggcagtc
MRE11	20	58	279	**TGTAAAACGACGGCCAGT**tgcccataagaacttctgtc	**CAGGAAACAGCTATGAC**TTATGGAGTTATGCTCAGGA
BLM	P1	58	696	**TGTAAAACGACGGCCAGT**GTCCATGGAGGCATCTGAGT	**CAGGAAACAGCTATGAC**CAGAGCAGGGCTAGATCAATG
BLM	P2	53	742	**TGTAAAACGACGGCCAGT**CCATTTATATGGATGAATGGAT	**CAGGAAACAGCTATGAC**TGTGTCTCTGCGATGTTTTG
BLM	CNS1	56	517	**TGTAAAACGACGGCCAGT**TTCCAGTGTCATCTACTGCTCAA	**CAGGAAACAGCTATGAC**CACATAAAACATGCTTCTCTACTTTG
BLM	CNS2	56	426	**TGTAAAACGACGGCCAGT**CCACTGAAGGCTCCAGAGAA	**CAGGAAACAGCTATGAC**GCTTCCTCAGGCTCTTCTTTC
BLM	CNS3	56	316	**TGTAAAACGACGGCCAGT**AAGCATTTTGGGTTTCTTCC	**CAGGAAACAGCTATGAC**CACCCAGCCTAATACGACAT
BLM	CNS4	56	435	**TGTAAAACGACGGCCAGT**CCGGCTACTTGGGAACCT	**CAGGAAACAGCTATGAC**GGGAATGAAGAAGAGAGAAGGTG
BLM	CNS5	58	507	**TGTAAAACGACGGCCAGT**AACAGAGTTGCTTGCAGAAAAA	**CAGGAAACAGCTATGAC**CAGGGTTGTCCTCCGATAAG
BLM	CNS6	58	418	**TGTAAAACGACGGCCAGT**AGTCCCTGTGCAGTGAAAGAA	**CAGGAAACAGCTATGAC**TTGAGGGTATAGGGCCACAG
BLM	2	50	313	**TGTAAAACGACGGCCAGT**agtccttcctcccctcaaaa	**CAGGAAACAGCTATGAC**catctgtacaatgggggagttt
BLM	3	58	588	**TGTAAAACGACGGCCAGT**tggattctttgctcagttgg	**CAGGAAACAGCTATGAC**TTTGGGGTGGTGTAACAAA
BLM	3	55	512	**TGTAAAACGACGGCCAGT**TCCCGGGATACTGCTCTC	**CAGGAAACAGCTATGAC**tcccaatggctagctttgaa
BLM	4	54	427	**TGTAAAACGACGGCCAGT**gccagaagcactcattctta	**CAGGAAACAGCTATGAC**tttttgaggctttcacttg
BLM	5	56	334	**TGTAAAACGACGGCCAGT**tgtctgatcagtggtagaaaa	**CAGGAAACAGCTATGAC**caatctttgtgttacgttgttt
BLM	6	57	315	**TGTAAAACGACGGCCAGT**ccatgcctagccaagacttt	**CAGGAAACAGCTATGAC**cattttgccctgtccatttt
BLM	7	54	533	**TGTAAAACGACGGCCAGT**atttgcttttgtggcctacc	**CAGGAAACAGCTATGAC**GGAAGGTTGGGTTTCTCTTT
BLM	7	54	535	**TGTAAAACGACGGCCAGT**TTTATTCAATACCCATTTACAGAAGTC	**CAGGAAACAGCTATGAC**tgatgatttgctatggtttttctta
BLM	8	58	428	**TGTAAAACGACGGCCAGT**gggcaagggaaatgctaaag	**CAGGAAACAGCTATGAC**caggaggtttaagaggtccctaa
BLM	9	61	335	**TGTAAAACGACGGCCAGT**agggacaatatgccttggtg	**CAGGAAACAGCTATGAC**aaaaggttatccagaggactgaaa
BLM	10	56	401	**TGTAAAACGACGGCCAGT**gatatgtatttcctttgataggtttga	**CAGGAAACAGCTATGAC**ttggggtttctggatgaaag
BLM	11	56	285	**TGTAAAACGACGGCCAGT**ccacagaatcatgaggtgatgt	**CAGGAAACAGCTATGAC**cactcagtgtgggtttttattca
BLM	12	52	321	**TGTAAAACGACGGCCAGT**cattgagcagtgttggcttt	**CAGGAAACAGCTATGAC**catgtagcagctctggcagt
BLM	13	54	351	**TGTAAAACGACGGCCAGT**tggggttaggattttaggg	**CAGGAAACAGCTATGAC**tgctgtcataatgcaaaaagg
BLM	14	56	430	**TGTAAAACGACGGCCAGT**gtgtgtggtcttccagcagt	**CAGGAAACAGCTATGAC**ccttcaaggtaaaaacagtttgc
BLM	15	50	383	**TGTAAAACGACGGCCAGT**ttatgaaaatgttccttcaagtc	**CAGGAAACAGCTATGAC**actggaccagaagtaccacaa
BLM	16	60	464	**TGTAAAACGACGGCCAGT**atgccatgttgacaatgctg	**CAGGAAACAGCTATGAC**tctgtcggagaccacctttt
BLM	17	45	325	**TGTAAAACGACGGCCAGT**accctagtaatctaggcatt	**CAGGAAACAGCTATGAC**tttctatgtacttgtaataaattc
BLM	18	58	428	**TGTAAAACGACGGCCAGT**tctgtccaaacctgtccataa	**CAGGAAACAGCTATGAC**tctataagcttggacaaagacact
BLM	19	54	434	**TGTAAAACGACGGCCAGT**ccccaaaaatgcaattaagc	**CAGGAAACAGCTATGAC**ccccaaaaagaaacaaacca
BLM	20	61	325	**TGTAAAACGACGGCCAGT**atgcgtgaatgagcctgaat	**CAGGAAACAGCTATGAC**gcattcacacaactgcatcc
BLM	21	54	432	**TGTAAAACGACGGCCAGT**atatggcagggaagcagcta	**CAGGAAACAGCTATGAC**aggcagagctgttgcacttt
BLM	22	60	407	**TGTAAAACGACGGCCAGT**cgtaggcagaaaatgcacaa	**CAGGAAACAGCTATGAC**AGTCACAGATGGTCAGATGCT
