# Supplementary material for: Genetic variation in the NBS1, MRE11, RAD50 and BLM genes and susceptibility to non-Hodgkin lymphoma
Source: BMC Med Genet. 2009 Nov 16;10:117. doi: 10.1186/1471-2350-10-117 (PMC2788526; doi:10.1186/1471-2350-10-117)
Supplement: Additional file 2 — Genotyping assays: primers, probes, and minor allele frequencies. Primers and probes used in genotyping assays, with genotyped minor allele frequencies. [file 1471-2350-10-117-S2.PDF]

Additional File 8 - Genotyping assays. Variant position in boldtype.

	Variant Name	VIC Probe*	6FAM Probe*	Forward Primer
5	RAD50_IVS4(+19)G/A	ACCCTTAAATA**G**ACTTTG	CCCTTAAATA**A**ACTTTG	GCTTTGAAGCAAAAGTTTGATGAGATTTT
8	RAD50_IVS7(-38)C/T	TTCAACT**G**GTGCTTAAA	TTTTTTCAACT**A**GTGCTTAAA	CGTGAATCTGCAGCTATCTCAACTT
24	RAD50_IVS22(+24)A/G	TGTATCACAA**A**TGCTCTTT	TATCACAA**G**TGCTCTTT	CGTGGACAAGGTGAGTACCAT
25	RAD50_IVS22(+62)A/G	TCTTCCCCTT**A**TGACCTC	CCCCTT**G**TGACCTC	CAAGGTGAGTACCATGGTGTATCA
29	NBS1_5(-905)T/C	CATGGCAAG**A**CCCTGT	ATGGCAAG**G**CCCTGT	CACCAAGCCCTGCTAACTTTAAAAT
35	NBS1_5UP_(-352)_del(AGTA)	ACATAACTGGTA**AGTA**TTT	ACATAACTGGTATTTTAAT	AAACTGCAAACGCGACGAAACT
36	NBS1_X2_(102)_G/A	TGCCATTCT**G**ATTGAA	TGTGCCATTCT**A**ATG	TTGAGTACGTTGTTGGAAGGAAAA
39	NBS1_IVS3(+208)G/A	CTTCATGGGAA**G**TTACA	TCATGGGAA**A**TTACA	AGTGGGCCCTGAGGGA
45	NBS1_X5_(553)_G/C	AGCAGTT**C**AGTCCAA	AGCAGTT**G**AGTCCAA	GACGTCCAATTGTAAAGCCAGAATA
59	NBS1_X13_(2016)_A/G	TTTACCTTACGTA**C**ATTTAG	TTTACCTTACGTA**T**ATTTAG	GGCTTTGTCATTGCATCTTTTTTTCATTTTT
64	NBS1_3UTR(+273)G/A	CCAGA**T**GGATTTCT	CCAGA**C**GGATTTC	TTGACTGAATTTAGATCACTGGTGATTAA
67	MRE11_5(-1703)A/G	CAGTAAGGTTTA**T**AGCCC	AGTAAGGTTTA**C**AGCCC	TCAACTCCTTACAAACAACTCAGTGAT
69	MRE11_5(-1456)C/T	CCGGCACT**C**TGCCAC	CGGCACT**T**TGCCAC	CTGGGAGGGAGGGAGAGG
72	MRE11_IVS2(+28)G/A	CTGGAATCCCTT**G**TTATT	CTGGAATCCCTT**A**TTATT	ATGCACTGTAAGCACCTGACTT
81	MRE11_IVS9(-60)A/T	CTTTCGT**T**TGCACATCA	CTTTCGT**A**TGCACATCA	GCAGAAACATTTATTTTTAAGAGTATGATTTTATAAAGAAGCA
95	BLM_IVS7(+388)C/T	CTTTGTATTAATC**G**ATTCTC	TTTGTATTAATC**A**ATTCTC	GCAAAAATGGATTCGCATGCTAAAAA
96	BLM_IVS7(+798)ins(T)	AGCAGCGTAA**A**CTG	CAGCAGCGTAACTG	ATTGATGACTTAATACCACGGAGGTTT
98	BLM_IVS12(+7)T/C	CGTGCAAC**A**ACTTA	ACGTGCAAC**G**ACTTA	TCCTGACTCAGCTGAAGATTCTCA
114	BLM_IVS21(-60)_del(GAA)	CAAAAATGAATGACCTTCCTC	ATGAATGACCTT**CTT**CCTC	TCCTTTATTCCATAAGTAGTAAAGGAAACTTACCT

Reverse Primer	Notes	Minor Allele Frequency
GCATCCAAATTGCAAACACAGTTCA		20.91%
TTGCAGCTGTAGACGACCTTTTAA	opposite strand	2.51%
TGCGGAGAGGGCTTTGG		4.73%
GGAGGTAATGCTGGCATGATGA		1.87%
GCCAGGAGTTCAAGATCAGCAT	opposite strand	34.73%
TAACTCCAAAGCAACATAAGGAAAATT		5.45%
AGCAGTTAACACAGCATGATTTCG		33.88%
CCTTCACCAATGAGGAATCGAAGA		39.32%
TTCAATTTGTGGAGGCTGCTT		33.82%
AATGGTGGAAGGGTGACTTTAGTC	opposite strand	33.15%
TGAAATTTTTTAGTTGACCATAATCATCA		32.91%
GTTCCATTGAAGGGTCCTTGATCT	opposite strand	33.29%
GCTCACGGGCCTGTGA		1.91%
CACAGTTGACGAGCTTTAGAAACC		44.29%
CACCGAGTCACAGTGTAAATTTCCT		32.34%
CATATCCTTTATAGACTACCCTATATTTTAACATTTTAACACAT	opposite strand	6.15%
ACACATTCTAACATGATACAAAAATAAATCCATTAATCG	opposite strand	4.82%
CCCCAGGGTTCTCAAATACGT	opposite strand	27.16%
CCCCCTGAAATGTGACAAAGAAAAA	opposite strand	34.56%
