# Supplementary material for: Genetic variation in the NBS1, MRE11, RAD50 and BLM genes and susceptibility to non-Hodgkin lymphoma
Source: BMC Med Genet. 2009 Nov 16;10:117. doi: 10.1186/1471-2350-10-117 (PMC2788526; doi:10.1186/1471-2350-10-117)
Supplement: Additional file 3 — Samples sequenced. Composition (gender, age and pathology) of the samples used in the re-sequencing phase. [file 1471-2350-10-117-S3.PDF]

Additional File 2 - Samples sequenced.

	No.	%
**Gender**		
Male	53	60.9%
Female	34	39.1%
**Age group (years)**		
20-24	4	4.6%
25-29	5	5.7%
30-34	8	9.2%
35-39	8	9.2%
40-44	21	24.1%
45-49	20	23.0%
50-54	21	24.1%
**Pathology**		
**B-cell lymphomas**		
DLBCL	29	33.3%
FL1	21	24.1%
FL2/FL3	9	10.3%
MZL/ MALT	4	4.6%
MCL	4	4.6%
SLL/CLL	2	2.3%
LPL	1	1.1%
MISC BCL	4	4.6%
**T-cell lymphomas**		
MF	6	6.9%
PTCL	3	3.4%
MISC TCL	1	1.1%
PTLD	2	2.3%
Unknown	1	1.1%
**Total**	87	

DLBCL = Diffuse Large B-Cell Lymphoma, FL = Follicular Lymphoma, FL1 = Follicular Lymphoma grade 1, FL2 = Follicular Lymphoma grade 2, FL3 = Follicular Lymphoma grade 3, MZ/MALT = Marginal Zone lymphoma/Mucosa-Associated Lymphoma Tissue lymphoma,MCL = Mantle Cell lymphoma, SLL = Small Lymphocytic Lymphoma, LPL = Lymphoplasmacytic Lymphoma, Misc. B-cell = Miscellaneous B-cell lymphoma, MF = Mycosis Fungoides, PTCL = Peripheral T-Cell Lymphoma, Misc. T-cell = Miscellaneous T-cell lymphoma.
