# Supplementary material for: Genetic variation in the NBS1, MRE11, RAD50 and BLM genes and susceptibility to non-Hodgkin lymphoma
Source: BMC Med Genet. 2009 Nov 16;10:117. doi: 10.1186/1471-2350-10-117 (PMC2788526; doi:10.1186/1471-2350-10-117)
Supplement: Additional file 4 — SNPs discovered by re-sequencing. Full list of all SNPs found by re-sequencing and their properties (chromosomal position, dbSNP number if known, flanking sequence, nucleotide/codon/amino acid change, and minor allele frequency). [file 1471-2350-10-117-S4.PDF]

Additional File 3 - SNPs discovered by re-sequencing.

	Variant Name	Chromosomal Position	dbSNP number, build 128	Flanking sequence	Nucleotide Change	Codon Change	Amino Acid	Minor Allele Frequency
1	RAD50_5UP_(-660)_T/C	5:131414499	rs2706335	agttttgaca[T/C]gaactgaccc	T/C	N/A	N/A	0.54%
2	RAD50_5UP_(-501)_G/A	5:131414657		gcaaagatcc[G/A]gaagtccgct	G/A	N/A	N/A	0.56%
3	RAD50_5UP_(-475)_A/C	5:131414682		tggcaacagc[A/C]cccagcacct	A/C	N/A	N/A	0.57%
4	RAD50_5UTR_(-38)_A/G	5:131415118	rs4526098	GCCTTTGTGG[G/A]CTCCAGGTCC	G/A	N/A	N/A	0.53%
**5**	**RAD50_IVS4_(+19)_G/A**	5:131437352	rs17166050	cccttaaata[G/A]actttgtagt	G/A	N/A	N/A	23.40%
6	RAD50_IVS6_(+11)_G/A	5:131445531		gtttgtggtg[G/A]tagaattttg	G/A	N/A	N/A	1.58%
7	RAD50_X7_(993)_G/A	5:131445847	rs28903091	GACTGTCATC[G/A]TGAACTGGAA	G/A	CGT/CAT	R/H	0.53%
**8**	**RAD50_IVS7_(-38)_C/T**	5:131446477		ttttaagcac[C/T]agttgaaaaa	C/T	N/A	N/A	2.72%
9	RAD50_IVS7_(-23)_T/C	5:131446491		gaaaaaaaaa[T/C]tatgagattt	T/C	N/A	N/A	0.54%
10	RAD50_X8_(1094)_G/A	5:131446555		GAACATATCC[G/A]AGCTAGAGAT	G/A	CGA/CAA	R/Q	0.54%
11	RAD50_X9_(1336)_A/G	5:131447546		AATTGAGTTA[A/G]AATCAGAAAT	A/G	AAA/GAA	***K/E***	0.53%
12	RAD50_X12_(1911)_T/A	5:131452810		GTAGCCAGGA[T/A]TTTGAAAGTG	T/A	GAT/GAA	D/E	0.53%
13	RAD50_IVS12_(+30)_A/C	5:131452897	rs2522390	ttatcaggat[A/C]ctttgacacc	A/C	N/A	N/A	0.53%
14	RAD50_X13_(2025)_C/T	5:131453449	rs34147298	AGCTAACAGA[C/T]GAAAACCAGT	C/T	GAC/GAT	D/D	0.53%
15	RAD50_X16_(2525)_T/C	5:131462625	rs28903093	ttttgtgtag[T/C]TTCTAGTAAG	T/C	GTT/GCT	V/A	0.53%
16	RAD50_IVS16_(-31)_A/G	5:131466402		atggaatatt[A/G]tataatactt	A/G	N/A	N/A	0.57%
17	RAD50_X18_(2840)_T/C	5:131466944		CTGAATGATA[T/C]AAAGAGAAGG	T/C	ATT/ACT	I/T	0.55%
18	RAD50_IVS18_(-5)_ins(T)	5:131467093		tttctttttt[-/T]gtagCAAAAA	-/T	N/A	N/A	0.53%
19	RAD50_IVS20_(-696)_T/A	5:131475188	rs2106984	ccaacaagac[T/A]tgtaatccag	T/A	N/A	N/A	9.09%
20	RAD50_IVS20_(-457)_del(AG)	5:131475426	rs33932778,rs2406542	atttttaata[AG/-]tattactgca	AG/-	N/A	N/A	27.85%
21	RAD50_IVS21_(-7628)_A/G	5:131487298	rs3798134	atttaggctt[G/A]ttaaggaaca	G/A	N/A	N/A	23.08%
22	RAD50_IVS21_(-7201)_C/T	5:131487724		tgtattaccc[C/T]atactaatac	C/T	N/A	N/A	23.37%
23	RAD50_IVS21_(-905)_C/T	5:131494019	rs12653750	ttcataaacc[C/T]gcttccaaag	C/T	N/A	N/A	22.16%
**24**	**RAD50_IVS22_(+24)_A/G**	5:131495032		tgtatcacaa[A/G]tgctctttcc	A/G	N/A	N/A	5.85%
**25**	**RAD50_IVS22_(+62)_A/G**	5:131495068	rs17166112	tcttcccctt[A/G]tgacctctca	A/G	N/A	N/A	3.23%
26	RAD50_IVS22_(+285)_A/G	5:131495290	rs2040704	gccaaaatgc[A/G]ataagaaaaa	A/G	N/A	N/A	24.18%
27	RAD50_IVS23_(+1205)_C/T	5:131497236		caatttaaag[C/T]atctgcagat	C/T	N/A	N/A	0.62%
28	RAD50_X25_(3858)_C/T	5:131500089		AGAAATTCTA[C/T]AGGATTAAAA	C/T	TAC/TAT	Y/Y	0.57%
**29**	**NBS1_5UP_(-905)_T/C**	8:89377795	rs13312842	aaagacaggg[T/C]cttgccatgt	T/C	N/A	N/A	34.18%
30	NBS1_5UP_(-810)_T/C	8:89377889	rs13312843	gagcgagcca[T/C]ggtgctgggc	T/C	N/A	N/A	2.47%
31	NBS1_5UP_(-775)_del(T)	8:89377923	rs13312844	ttaaagtgac[T/-]tttcccccct	T/-	N/A	N/A	0.62%
32	NBS1_5UP_(-732)_G/A	8:89377965	rs13312846	caaaatgtat[G/A]tgatcaggag	G/A	N/A	N/A	1.30%
33	NBS1_5UP_(-550)_G/A	8:89378146	rs1805800	ccagtattta[G/A]catgtgatta	G/A	N/A	N/A	32.54%
34	NBS1_5UP_(-425)_T/C	8:89378270		atcattatta[T/C]caccattact	T/C	N/A	N/A	0.69%
**35**	**NBS1_5UP_(-352)_del(AGTA)**	8:89378342	rs36226237	ataactggta[A/G GTAdel]ttttaatgca	A/G GTAdel	N/A	N/A	5.63%
**36**	***NBS1_X2_(102)_G/A***	8:89380460	rs1063045	GTGCCATTCT[G/A]ATTGAAAATG	G/A	CTG/CTA	L/L	32.89%
37	NBS1_IVS2_(-541)_del(G)	8:89381186		tttaatcaaa[G/-]attgcatata	G/-	N/A	N/A	0.57%
38	NBS1_IVS2_(-448)_A/G	8:89381278		atttctacag[A/G]aaaagtaaaa	A/G	N/A	N/A	0.57%
**39**	**NBS1_IVS3_(+208)_G/A**	8:89382081	rs1805796	ttcatgggaa[G/A]ttacatttct	G/A	N/A	N/A	40.00%
40	NBS1_IVS3_(-224)_T/C	8:89382130		taacttttgg[T/C]cattgttttt	T/C	N/A	N/A	0.53%
41	NBS1_IVS3_(-159)_del(ATT)	8:89382194		tacaaagctt[AAT/-]gatgaggaac	AAT/-	N/A	N/A	0.53%

	Variant Name	Chromosomal Position	dbSNP number, build 128	Flanking sequence	Nucleotide Change	Codon Change	Amino Acid	Minor Allele Frequency
42	NBS1_IVS3_(-29)_G/A	8:89382321	rs769417	ctgatactat[G/A]actttattta	G/A	N/A	N/A	0.53%
43	NBS1_X4_(381)_T/C	8:89382409		GGAAAACTGC[T/C]TTAAATCAAG	T/C	GCT/GCC	A/G	0.53%
44	NBS1_IVS4_(+292)_C/G	8:89382799	rs1805841	acagtgcata[C/G]tttgtgtttt	C/G	N/A	N/A	40.34%
**45**	***NBS1_X5_(553)_G/C***	8:89384989	rs1805794	GAAAGCAGTT[G/C]AGTCCAAGAA	G/C	GAG/GAC	E/D	34.21%
46	NBS1_IVS5_(+327)_G/A	8:89385346	rs1805834	agttagctct[G/A]gaattgtgtg	G/A	N/A	N/A	0.53%
47	NBS1_X6_(643)_C/T	8:89392006	rs34767364	TCTGTCAGGA[C/T]GGCAGGAAAG	C/T	CGG/TGG	***R/W***	0.54%
48	NBS1_X6_(657)_del(ACAAA)	8:89392016		CGGCAGGAAA[AACA/-]AAAACAAATC	AACA/-	frameshift	frameshift	0.54%
49	NBS1_IVS6_(-29)_C/T	8:89392645		ctacatttta[C/T]tttaaaaaat	C/T	N/A	N/A	1.70%
50	NBS1_IVS6_(-18)_G/A	8:89392655	rs769418	tttaaaaaat[G/A]tttccttcca	G/A	N/A	N/A	3.98%
51	NBS1_IVS7_(+36)_G/A	8:89392901	rs1805826	aaaactgcaa[G/A]taggagattt	G/A	N/A	N/A	4.02%
52	NBS1_IVS7_(-64)_A/G	8:89398656	rs13312891	ttatcttgac[A/G]ttatctgaat	A/G	N/A	N/A	0.52%
53	NBS1_IVS7_(-42)_G/C	8:89398677		aaagttgcta[G/C]ttttaatata	G/C	N/A	N/A	2.06%
54	NBS1_IVS9_(+18)_C/T	8:89404518	rs2234744	ttcttcatta[C/T]cgtactattg	C/T	N/A	N/A	36.26%
55	NBS1_IVS9_(+91)_C/A	8:89404590	rs1805818	caggttgtct[C/A]ttgagagaga	C/A	N/A	N/A	36.81%
56	NBS1_X10_(1197)_T/C	8:89407740	rs709816	TTTCACAAGA[T/C]GCACCCACTG	T/C	GAT/GAC	D/D	41.86%
57	NBS1_X11_(1718)_T/A	8:89409853		AAAACCAGAG[T/A]TAGAAATTGA	T/A	TTA/AAA	***L/K***	0.56%
58	NBS1_IVS12_(-7)_A/G	8:89416919	rs2308962	tcttaacccc[A/G]tttaagAACA	A/G	N/A	N/A	35.23%
**59**	***NBS1_X13_(2016)_A/G***	8:89417026	rs1061302	CCAGAAATCC[A/G]TCTGGCATAA	A/G	CCA/CCG	P/P	34.27%
60	NBS1_IVS13_(-61)_A/T	8:89419792	rs3736640	taatcttttt[A/T]aaaaaatggt	A/T	N/A	N/A	2.81%
61	NBS1_IVS13_(-30)_A/T	8:89419822	rs3736639	tgttaatatg[A/T]tttactgtac	A/T	N/A	N/A	34.83%
62	NBS1_X14_(2082)_T/G	8:89419862	rs7823648	TCACATATCC[T/G]GGAGCAGGAA	T/G	CCT/CCG	P/P	0.56%
63	NBS1_IVS15_(+88)_C/G	8:89426278	rs13312970	tttgtttctt[C/G]tgtcctcata	C/G	N/A	N/A	3.26%
**64**	**NBS1_3UTR_(+273)_G/A**	8:89427902	rs1063053	AATTCTAAAT[A/G]TACGTAAGGT	A/G	N/A	N/A	35.47%
65	MRE11_5UP_(-2025)_C/A	11:96479526	rs13447576	aatatagtaa[C/A]cttataccac	C/A	N/A	N/A	2.84%
66	MRE11_5UP_(-1912)_T/G	11:96479638	rs13447578	tgtacaaaat[T/G]ttactggtat	T/G	N/A	N/A	0.57%
**67**	**MRE11_5UP_(-1703)_A/G**	11:96479846	rs11020803	aggcggggct[A/G]taaaccttac	A/G	N/A	N/A	32.76%
68	MRE11_5UP_(-1510)_C/T	11:96480038	rs13447579	ggggcgacac[C/T]tgtagagggt	C/T	N/A	N/A	0.57%
**69**	**MRE11_5UP_(-1456)_C/T**	11:96480091	rs13447580	ctccggcact[C/T]tgccaccagc	C/T	N/A	N/A	3.45%
70	MRE11_5UP_(-1158)_G/T	11:96480388		tggctcctgc[G/T]tgagggcggg	G/T	N/A	N/A	29.31%
71	MRE11_5UP_(-1131)_C/A	11:96480414	rs12708334	ccgctacttt[C/A]cccgcccccct	C/A	N/A	N/A	1.15%
**72**	**MRE11_IVS2_(+28)_G/A**	11:96481591	rs497763	ggaatccctt[G/A]ttatttgggg	G/A	N/A	N/A	39.78%
73	MRE11_IVS3_(-143)_G/C	11:96483236	rs1009455	gaatacttca[G/C]attttcagat	G/C	N/A	N/A	1.08%
74	MRE11_IVS3_(-87)_del_ATT	11:96483291		aactgttatt[ATT/-]tattttataa	ATT/-	N/A	N/A	0.53%
75	MRE11_IVS4_(-33)_A/G	11:96488223		cctgtaaaac[A/G]tactaatttt	A/G	N/A	N/A	0.53%
76	MRE11_IVS6_(-294)_A/G	11:96495170	rs13447611	gttgcttaat[A/G]ttacaaagca	A/G	N/A	N/A	0.57%
77	MRE11_IVS6_(-146)_A/G	11:96495317		tacttttact[A/G]ttgtaatttt	A/G	N/A	N/A	0.57%
78	MRE11_IVS6_(-112)_T/C	11:96495350	rs680695	actatcctca[T/C]ctatgttttt	T/C	N/A	N/A	31.82%
79	MRE11_IVS6_(-6)_G/A	11:96495456	rs535801	aaattatttt[G/A]catagGCAGA	G/A	N/A	N/A	30.53%
80	MRE11_X9_(822)_T/C	11:96502741		TTACTTCTCT[T/C]TCCCCAGGAG	T/C	CTT/CTC	L/L	0.53%
**81**	**MRE11_IVS9_(-60)_A/T**	11:96503635	rs610611	aagctttcgt[A/T]tgcacatcac	A/T	N/A	N/A	35.80%
82	MRE11_IVS11_(+17)_T/C	11:96506540	rs1805365	cctatttata[T/C]agattgaata	T/C	N/A	N/A	1.08%
83	MRE11_IVS12_(+19)_T/C	11:96510241	rs641936	gttattttta[T/C]gaggtaaaaa	T/C	N/A	N/A	32.76%
84	MRE11_X14_(1475)_C/A	11:96514901		CATATTGATG[C/A]CCTCGAAGAC	C/A	GCC/GAC	A/D	0.54%

	Variant Name	Chromosomal Position	dbSNP number, build 128	Flanking sequence	Nucleotide Change	Codon Change	Amino Acid	Minor Allele Frequency
85	BLM_5UP_(-30523)_C/T	15:88847333	rs28364254	gcccactttc[C/T]cggttcaatg	C/T	N/A	N/A	1.08%
86	BLM_5UP_(-30397)_G/C	15:88847458	rs28364255	ggaaccaaga[G/C]aatggggtgc	G/C	N/A	N/A	0.54%
87	BLM_IVS1_(-29936)_G/C	15:88847918		taccgcgcgc[G/C]taactacggg	G/C	N/A	N/A	0.54%
88	BLM_X2_(11)_T/C	15:88877863		ATGGCTGCTG[T/C]TCCTCAAAAT	T/C	GTT/GCT	V/A	0.63%
89	BLM_IVS2_(+6)_T/G	15:88877955		TTTTCgtaag[T/G]gttttgactg	T/G	N/A	N/A	0.63%
90	BLM_IVS2_(+33)_G/T	15:88877981	rs28384975	tgtcacatag[G/T]cactaactta	G/T	N/A	N/A	0.63%
91	BLM_X4_(893)_C/T	15:88882337	rs28384991	GATTATGATA[C/T]GGATTTTGTT	C/T	ACG/ATG	T/M	0.56%
92	BLM_X5_(968)_A/G	15:88885275		agTACGTTAA[A/G]GGACCTTGAC	A/G	AAG/AGG	K/R	0.60%
93	BLM_X7_(1481)_C/T	15:88891309		TTATTCAATA[C/T]CCATTTACAG	C/T	ACC/AGC	T/S	0.79%
94	BLM_X7_(1722)_A/G	15:88891549	rs28385011	TGCATAATTT[A/G]GCAGCCAGCA	A/G	TTA/TTG	L/L	0.60%
**95**	**BLM_IVS7_(+388)_C/T**	15:88892096	rs28385015	tgatgagaat[C/T]gattaataca	C/T	N/A	N/A	5.85%
**96**	**BLM_IVS7_(+798)_ins(T)**	15:88892506	rs28385016	tcttacagtt[T/-]acgctgctga	T/-	N/A	N/A	5.79%
97	BLM_X8_(2049)_A/G	15:88893584		TGCTTGGTGA[A/G]GACTGTTTTA	A/G	GAA/GAG	E/E	0.58%
**98**	**BLM_IVS12_(+7)_T/C**	15:88900044	rs3815003	AGGTgtaagt[T/C]gttgcacgtc	T/C	N/A	N/A	28.26%
99	BLM_IVS12_(+1397)_A/G	15:88901433		ccagcctaga[A/G]ttattttatt	A/G	N/A	N/A	0.56%
100	BLM_X13_(2603)_C/T	15:88914712	rs11852361	TATGTATTAC[C/T]GAAAAAGCCT	C/T	CCG/CTG	***P/L***	5.79%
101	BLM_IVS13_(+53)_A/C	15:88914823		tctttttagt[A/C]ccacaataag	A/C	N/A	N/A	0.53%
102	BLM_X16_(3102)_G/A	15:88926090	rs2227933	AAAATATAAC[G/A]GAATGCAGGA	G/A	ACG/ACA	T/T	13.01%
103	BLM_IVS16_(+44)_del(T)	15:88926242		aaattttttt[T/-]ctcttacttt	T/-	N/A	N/A	14.56%
104	BLM_X18_(3532)_C/A	15:88935533	rs2227934	GAAATAAAGC[C/A]CAAACTGTAC	C/A	GCC/GCA	A/A	12.63%
105	BLM_IVS19_(+2137)_G/T	15:88938335		gggcgacaga[G/T]cgagactcca	G/T	N/A	N/A	16.32%
106	BLM_IVS19_(+2339)_C/T	15:88938536		tcctggaaaa[C/T]aacgtcttca	C/T	N/A	N/A	1.05%
107	BLM_IVS19_(+2368)_C/A	15:88938564		gccagctggg[C/A]tatattacct	C/A	N/A	N/A	3.16%
108	BLM_X21_(3945)_C/T	15:88941111	rs1063147	CTGAGGAGCT[C/T]GACGAGGAAA	C/T	CTC/CTT	L/L	5.91%
109	BLM_X21_(3961)_G/A	15:88941126	rs7167216	GGAAATACCC[G/A]TATCTTCCCA	G/A	GTA/ATA	V/I	16.85%
110	BLM_IVS21_(+1248)_del(CA)	15:88942488		aaaaagtcca[CA/-]gtcaactttt	CA/-	N/A	N/A	15.34%
111	BLM_IVS21_(+1370)_C/T	15:88942608	rs28385166	accaaattgt[C/T]atttttcttgt	C/T	N/A	N/A	4.49%
112	BLM_IVS21_(+1453)_G/A	15:88942690	rs28385167	attagaaaac[G/A]ttagtttaat	G/A	N/A	N/A	4.35%
113	BLM_IVS21_(+1617)_C/A	15:88942853	rs414634	ctcaacaccc[C/A]gggtttttga	C/A	N/A	N/A	26.34%
**114**	**BLM_IVS21_(-60)_del(GAA)**	15:88944874	rs10685387	ttgagaggaa[GAA/-]ggtcattcat	GAA/-	N/A	N/A	33.89%

SNPs that were also genotyped marked in bold print. Chromosomal positions from the National Centre for Biotechnology Information Human Genome build 36. Amino acids marked in bold italic print are designated as "possibly damaging/probably damaging" by Polyphen software (see text for reference).
