# Supplementary figures and images for: Genetic variation in the NBS1, MRE11, RAD50 and BLM genes and susceptibility to non-Hodgkin lymphoma
Source: BMC Med Genet. 2009 Nov 16;10:117. doi: 10.1186/1471-2350-10-117 (PMC2788526; doi:10.1186/1471-2350-10-117)

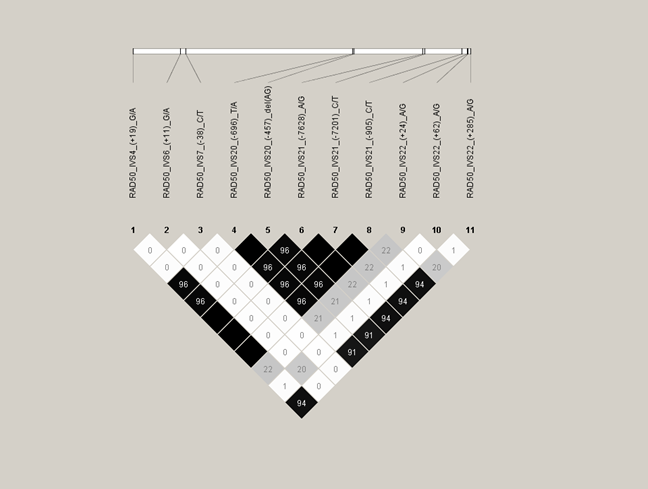

Supplement: Additional file 5 — Linkage disequilibrium between SNPs in RAD50. Correlations between SNPs in RAD50 as measured by r2 values. [file 1471-2350-10-117-S5.PNG]

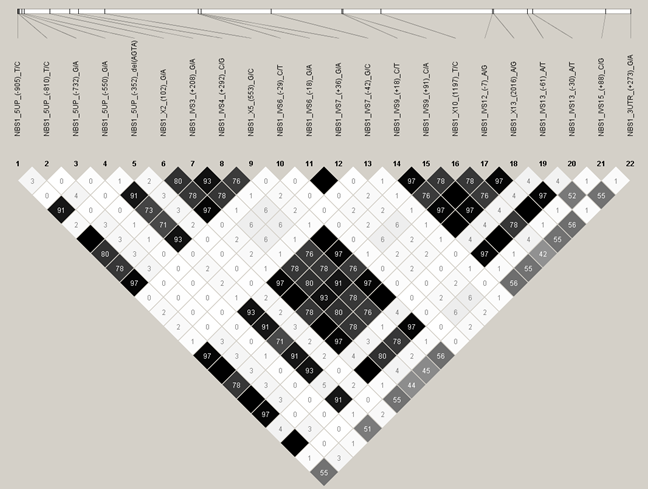

Supplement: Additional file 6 — Linkage disequilibrium between SNPs in NBS1. Correlations between SNPs in NBS1 as measured by r2 values. [file 1471-2350-10-117-S6.PNG]

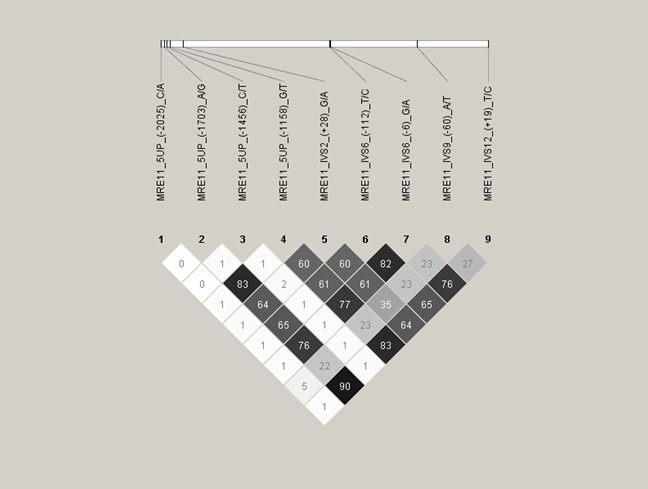

Supplement: Additional file 7 — Linkage disequilibrium between SNPs in MRE11. Correlations between SNPs in MRE11 as measured by r2 values. [file 1471-2350-10-117-S7.PNG]

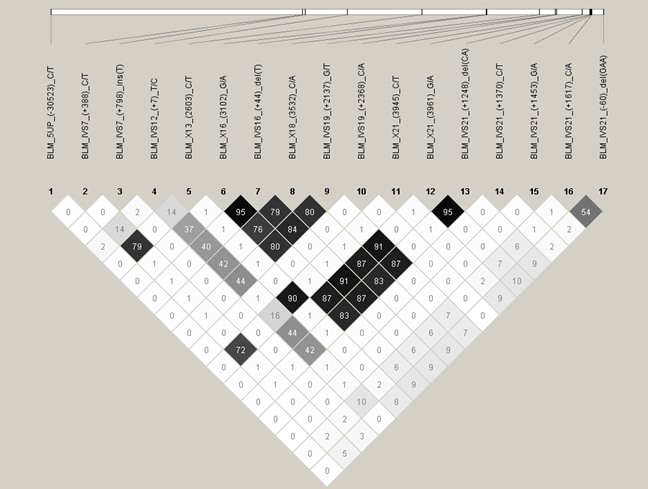

Supplement: Additional file 8 — Linkage disequilibrium between SNPs in BLM. Correlations between SNPs in BLM as measured by r2 values. [file 1471-2350-10-117-S8.PNG]
