# Supplementary material for: Genetic variation in the NBS1, MRE11, RAD50 and BLM genes and susceptibility to non-Hodgkin lymphoma
Source: BMC Med Genet. 2009 Nov 16;10:117. doi: 10.1186/1471-2350-10-117 (PMC2788526; doi:10.1186/1471-2350-10-117)
Supplement: Additional file 9 — Regression analysis in Caucasian samples. Table containing statistical analysis results in Caucasian samples, for overall NHL and all subtypes examined, with the following sections: Additional file 9a: Regression analysis results for RAD50 SNPs in Caucasian samples. Additional file 9b: Regression analysis results for NBS1 SNPs in Caucasian samples. Additional file 9c: Regression analysis results for MRE11 SNPs in Caucasian samples. Additional file 9d: Regression analysis results for BLM SNPs in Caucasian samples. [file 1471-2350-10-117-S9.PDF]

Additional File 9.1: Regression analysis for RAD50 SNPs in Caucasian samples only.

	Controls	All NHL			All B-cell NHL (with DLBC & FL)			DLBCL		
**SNP**	**N**	**N**	**OR (95% CI)**	**p value**	**N**	**OR (95% CI)**	**p value**	**N**	**OR (95% CI)**	**p value**
**RAD50_IVS4(+19)G/A**										
G/G	384	394	1	*0.991*	357	1	*0.784*	100	1	*0.896*
G/A	187	191	1.00 (0.78 - 1.28)	0.997	181	1.05 (0.81 - 1.35)	0.729	53	1.05 (0.72 - 1.54)	0.799
A/A	27	28	1.00 (0.58 - 1.74)	0.988	26	1.02 (0.58 - 1.78)	0.960	7	0.96 (0.40 - 2.30)	0.935
**RAD50_IVS7(-38)C/T**										
C/C	568	580	1	-	536	1	-	153	1	-
C/T	34	38	1.14 (0.71 - 1.85)	0.585	33	1.08 (0.66 - 1.78)	0.766	9	1.01 (0.47 - 2.17)	0.983
T/T	1	2	2.17 (0.19 - 24.40)	0.532	2	2.34 (0.21 - 26.32)	0.491	0	0.00 (0.00 -)	1.000
C/T & T/T	35	40	1.17 (0.73 - 1.88)	0.510	35	1.11 (0.68 - 1.82)	0.665	9	0.97 (0.45 - 2.08)	0.941
**RAD50_IVS22(+24)A/G**										
A/A	546	545	1	-	500	1	-	137	1	**-**
A/G	54	75	1.40 (0.96 - 2.03)	0.080	71	1.43 (0.98 - 2.09)	0.062	25	1.77 (1.05 - 2.96)	**0.031**
G/G	1	1	1.22 (0.08 - 10.01)	0.887	1	1.32 (0.08 - 21.54)	0.848	1	4.54 (0.26 - 78.43)	0.298
A/G & G/G	55	76	1.39 (0.96 - 2.02)	0.079	72	1.43 (0.98 - 2.08)	0.062	26	1.81 (1.09 - 3.01)	**0.022**
**RAD50_IVS22(+62)A/G**										
A/A	601	617	-	-	569	-	-	161	-	-
A/G	2	2	-	-	2	-	-	2	-	-
G/G	0	0	-	-	0	-	-	0	-	-
	**Controls**	**FL**			**MZ/MALT**			**MCL**		
**SNP**	**N**	**N**	**OR (95% CI)**	**p value**	**N**	**OR (95% CI)**	**p value**	**N**	**OR (95% CI)**	**p value**
**RAD50_IVS4(+19)G/A**										
G/G	384	107	1	*0.474*	43	1	-	32	1	-
G/A	187	55	1.08 (0.74 - 1.58)	0.677	19	1.01 (0.57 - 1.80)	0.975	11	1.23 (0.36 - 4.29)	0.740
A/A	27	10	1.31 (0.61 - 2.81)	0.492	3	1.03 (0.29 - 3.61)	0.966	0	0.00 (0.00 -)	1.000
G/A & A/A	214				22	1.01 (0.58 - 1.76)	0.967	11	0.61 (0.30 - 1.26)	0.182
**RAD50_IVS7(-38)C/T**										
C/C	568	166	1	-	58	1	**-**			
C/T	34	6	0.63 (0.26 - 1.53)	0.304	8	3.02 (1.28 - 7.14)	**0.012**	40	-	-
T/T	1	1	3.41 (0.21 - 56.13)	0.391	1	24.77 (1.43 - 427.94)	**0.027**	3	-	-
C/T & T/T	35	7	0.71 (0.31 - 1.64)		9	3.39 (1.48 - 7.75)	**0.004**	0	-	-
**RAD50_IVS22(+24)A/G**										
A/A	546	150	1	-	59	1	-	41	-	-
A/G	54	24	1.61 (0.96 - 2.71)	0.073	7	1.30 (0.55 - 3.04)	0.550	2	-	-
G/G	1	0	0.00 (0.00 -)	1.000	0	0.00 (0.00 -)	1.000	0	-	-
A/G & G/G	55	24	1.59 (0.94 - 2.67)	0.081	7	1.29 (0.55 - 3.02)	0.560			
**RAD50_IVS22(+62)A/G**										
A/A	601	174	-	-	67	-	-	43	-	-
A/G	2	0	-	-	0	-	-	0	-	-
G/G	0	0	-	-	0	-	-	0	-	-
	**Controls**	**SLL**			**LPL**			**Misc. B-cell**		
**SNP**	**N**	**N**	**OR (95% CI)**	**p value**	**N**	**OR (95% CI)**	**p value**	**N**	**OR (95% CI)**	**p value**
**RAD50_IVS4(+19)G/A**										
G/G	384	21	1	-	21	1	-	33	1	-
G/A	187	13	1.27 (0.61 - 2.63)	0.520	13	1.31 (0.63 - 2.70)	0.469	17	1.10 (0.59 - 2.04)	0.775
A/A	27	2	1.33 (0.29 - 6.15)	0.720	2	1.50 (0.33 - 6.84)	0.603	2	0.86 (0.19 - 3.83)	0.844
G/A & A/A	214	15	1.28 (0.64 - 2.56)	0.492	15	1.33 (0.67 - 2.66)	0.420	19	1.06 (0.59 - 1.94)	0.838
**RAD50_IVS7(-38)C/T**										
C/C	568	36	-	-	33	-	-	50	-	-
C/T	34	1	-	-	3	-	-	3	-	-
T/T	1	0	-	-	0	-	-	0	-	-
C/T & T/T	35									
**RAD50_IVS22(+24)A/G**										
A/A	546	33	-	-	30	1	-	50	-	-
A/G	54	4	-	-	6	2.13 (0.84 - 5.42)	0.113	3	-	-
G/G	1	0	-	-	0	0.00 (0.00 -)	1.000	0	-	-
A/G & G/G	55				6	2.10 (0.82 - 5.33)	0.120			
**RAD50_IVS22(+62)A/G**										
A/A	601	36	-	-	36	-	-	52	-	-
A/G	2	0	-	-	0	-	-	0	-	-
G/G	0	0	-	-	0	-	-	0	-	

SNP	Controls	All T-cell NHL			MF			PTCL		
	N	N	OR (95% CI)	p value	N	OR (95% CI)	p value	N	OR (95% CI)	p value
**RAD50_IVS4(+19)G/A**										
G/G	384	37	1	-	21	1	-	14	-	-
G/A	187	10	0.54 (0.26 - 1.12)	0.098	7	0.65 (0.27 - 1.59)	0.347	2	-	-
A/A	27	2	0.68 (0.15 - 2.99)	0.605	0	0.00 (0.00 -)	0.998	2	-	-
G/A & A/A	214	12	0.56 (0.28 - 1.11)	0.094	7	0.56 (0.23 - 1.35)	0.198			
**RAD50_IVS7(-38)C/T**										
C/C	568	44	1	-	24	-	-	17	-	-
C/T	34	5	2.20 (0.80 - 6.06)	0.127	4	-	-	1	-	-
T/T	1	0	0.00 (0.00 -)	1.000	0	-	-	0	-	-
C/T & T/T	35	5	2.11 (0.77 - 5.80)	0.146						
**RAD50_IVS22(+24)A/G**										
A/A	546	45	-	-	26	-	-	17	-	-
A/G	54	4	-	-	2	-	-	1	-	-
G/G	1	0	-	-	0	-	-	0	-	-
A/G & G/G	55									
**RAD50_IVS22(+62)A/G**										
A/A	601	48	-	-	28	-	-	18	-	-
A/G	2	0	-	-	0	-	-	0	-	-
G/G	0	0	-	-	0	-	-	0	-	-

OR = Odds Ratio, CI = Confidence Interval, NHL = Non-Hodgkin Lymphoma, DLBCL = Diffuse Large B-Cell Lymphoma, FL = Follicular Lymphoma, MZ/MALT = Marginal Zone lymphoma/Mucosa-Associated Lymphoma Tissue lymphoma,MCL = Mantle Cell lymphoma, SLL = Small Lymphocytic Lymphoma, LPL=Lymphoplasmacytic Lymphoma, Misc. B-cell = Miscellaneous B-cell lymphoma, MF = Mycosis Fungoides, PTCL = Peripheral T-Cell Lymphoma.

If less than 5 samples were in a category, the analysis is not valid and marked by "-". Analyses were not done for subtypes that had fewer than 5 heterozygotes and minor homozygotes combined. Analysis is adjusted for adjusted for gender, ethnicity, age, and residence.

p-value for test for trend is shown in italic type.

p-values less than 0.05 are in bold.

Additional File 9.2: Regression analysis for NBS1 SNPs in Caucasian samples only.

	Controls	All NHL			All B-cell NHL (with DLBC & FL)			DLBCL		
**SNP**	**N**	**N**	**OR (95% CI)**	**p value**	**N**	**OR (95% CI)**	**p value**	**N**	**OR (95% CI)**	**p value**
**NBS1_5(-905)T/C**										
T/T	266	286	1	*0.830*	266	1	*0.719*	76	1	*0.465*
T/C	267	266	0.93 (0.73 - 1.18)	0.557	242	0.91 (0.71 - 1.16)	0.438	72	0.95 (0.66 - 1.37)	0.769
C/C	64	69	1.02 (0.69 - 1.49)	0.929	63	1.00 (0.68 - 1.48)	0.986	14	0.77 (0.41 - 1.46)	0.423
**NBS1_5UTR_(-352)_del(AGTA)**										
AGTA/AGTA	524	519	1	-	475	1	-	138	1	-
AGTA/-	58	57	0.99 (0.67 - 1.46)	0.946	54	1.02 (0.69 - 1.52)	0.917	16	1.06 (0.59 - 1.92)	0.839
-/-	2	3	1.61 (0.26 - 9.77)	0.606	3	1.74 (0.29 - 10.61)	0.548	0	0.00 (0.00 -)	0.999
AGTA/- & -/-	60	60	1.01 (0.69 - 1.48)	0.973	57	1.04 (0.71 - 1.54)	0.826	16	1.03 (0.57 - 1.85)	0.929
**NBS1_IVS3(+208)G/A**										
G/G	241	266	1	*0.615*	248	1	*0.543*	66	1	*0.672*
G/A	277	266	0.87 (0.68 - 1.11)	0.266	241	0.85 (0.66 - 1.09)	0.187	76	1.00 (0.68 - 1.45)	0.977
A/A	78	85	0.99 (0.69 - 1.41)	0.939	79	0.98 (0.68 - 1.41)	0.915	19	0.86 (0.48 - 1.52)	0.596
**NBS1_3UTR(+273)G/A**										
G/G	268	288	1	*0.496*	267	1	*0.536*	78	1	*0.256*
G/A	263	263	0.95 (0.74 - 1.20)	0.652	237	0.92 (0.72 - 1.17)	0.481	73	0.97 (0.68 - 1.40)	0.885
A/A	63	60	0.88 (0.60 - 1.31)	0.533	58	0.93 (0.62 - 1.38)	0.713	11	0.60 (0.24 - 1.19)	0.142
**NBS1_X2_(102)_G/A**										
G/G	266	285	1	*0.818*	265	1	*0.718*	75	1	*0.521*
G/A	272	270	0.93 (0.73 - 1.18)	0.559	245	0.91 (0.71 - 1.16)	0.433	72	0.95 (0.66 - 1.37)	0.782
A/A	59	63	1.02 (0.69 - 1.51)	0.927	58	1.01 (0.68 - 1.51)	0.959	13	0.79 (0.41 - 1.53)	0.486
**NBS1_X5_(553)_G/C**										
G/G	255	282	1	*0.522*	262	1	*0.437*	76	1	*0.306*
G/C	270	271	0.91 (0.72 - 1.16)	0.456	246	0.89 (0.70 - 1.14)	0.353	72	0.90 (0.62 - 1.30)	0.572
C/C	57	58	0.292 (0.619 - 1.395)	0.723	53	0.92 (0.61 - 1.39)	0.681	12	0.70 (0.36 - 1.39)	0.310
**NBS1_X13_(2016)_A/G**										
A/A	247	276	1	*0.339*	256	1	*0.305*	74	1	*0.204*
A/G	265	260	0.88 (0.69 - 1.13)	0.323	236	0.86 (0.67 - 1.11)	0.249	70	0.89 (0.61 - 1.30)	0.549
G/G	55	54	0.88 (0.58 - 1.33)	0.540	50	0.88 (0.58 - 1.35)	0.555	10	0.61 (0.29 - 1.26)	0.180

	Controls	FL			MZ/MALT			MCL		
**SNP**	**N**	**N**	**OR (95% CI)**	**p value**	**N**	**OR (95% CI)**	**p value**	**N**	**OR (95% CI)**	**p value**
**NBS1_5(-905)T/C**										
T/T	266	87	1	*0.847*	28	1	*0.816*	266	1	*0.673*
T/C	267	63	0.75 (0.51 - 1.08)	0.119	43	1.12 (0.65 - 1.93)	0.678	267	0.96 (0.49 - 1.89)	0.909
C/C	64	24	1.20 (0.70 - 2.06)	0.498	7	1.02 (0.42 - 2.48)	0.964	64	1.37 (0.52 - 3.63)	0.529
**NBS1_5UTR_(-352)_del(AGTA)**										
AGTA/AGTA	524	137	1	-	60	1	-	34	1	-
AGTA/-	58	18	1.08 (0.61 - 1.92)	0.785	2	0.30 (0.07 - 1.29)	0.105	6	1.55 (0.61 - 3.94)	0.358
-/-	2	3	5.57 (0.90 - 34.52)	0.065	0	0.00 (0.00 -)	0.999	0	0.00 (0.00 -)	0.999
AGTA/- & -/-	60	21	1.23 (0.71 - 2.11)	0.461	2	-	-	6	1.48 (0.59 - 3.77)	0.406
**NBS1_IVS3(+208)G/A**										
G/G	241	84	1	*0.466*	26	1	*0.818*	18	1	*0.707*
G/A	277	63	0.67 (0.46 - 0.97)	**0.033**	30	0.97 (0.56 - 1.71)	0.935	17	0.84 (0.42 - 1.68)	0.621
A/A	78	27	1.04 (0.62 - 1.72)	0.892	10	1.14 (0.52 - 2.51)	0.744	8	1.35 (0.56 - 3.28)	0.503
**NBS1_3UTR(+273)G/A**										
G/G	268	90	1	*0.509*	26	1	*0.807*	21	1	*0.605*
G/A	263	54	0.65 (0.44 - 0.95)	**0.026**	33	1.28 (0.74 - 2.23)	0.381	14	0.68 (0.33 - 1.38)	0.283
A/A	63	24	1.12 (0.66 - 1.91)	0.669	6	0.89 (0.35 - 2.29)	0.813	8	1.75 (0.73 - 4.22)	0.210
**NBS1_X2_(102)_G/A**										
G/G	266	87	1	*0.935*	28	1	*0.698*	19	1	*0.784*
G/A	272	62	0.72 (0.50 - 1.04)	0.082	33	1.15 (0.67 - 1.97)	0.615	19	0.99 (0.51 - 1.94)	0.983
A/A	59	24	1.31 (0.76 - 2.25)	0.326	7	1.09 (0.45 - 2.67)	0.849	5	1.23 (0.44 - 3.50)	0.694
**NBS1_X5_(553)_G/C**										
G/G	255	84	1	*0.786*	28	1	*0.938*	19	1	*0.847*
G/C	270	62	0.73 (0.50 - 1.06)	0.098	33	1.12 (0.65 - 1.92)	0.688	19	0.96 (0.49 - 1.88)	0.909
C/C	57	22	1.22 (0.70 - 2.13)	0.486	6	0.92 (0.36 - 2.36)	0.857	5	1.20 (0.42 - 3.41)	0.731
**NBS1_X13_(2016)_A/G**										
A/A	247	83	1	*0.634*	27	1	*0.897*	19	1	*0.939*
A/G	265	59	0.69 (0.47 - 1.01)	0.055	33	1.13 (0.65 - 1.95)	0.671	17	0.86 (0.43 - 1.71)	0.671
G/G	55	21	1.20 (0.68 - 2.12)	0.537	6	0.94 (0.36 - 2.43)	0.902	5	1.24 (0.43 - 3.52)	0.692

SNP	Controls N	SLL N	SLL OR (95% CI)	SLL p value	LPL N	LPL OR (95% CI)	LPL p value	Misc. B-cell N	Misc. B-cell OR (95% CI)	Misc. B-cell p value
**NBS1_5(-905)T/C**										
T/T	266	12	1	*0.196*	22	1	-	22	1	-
T/C	267	19	1.60 (0.75 - 3.40)	0.223	11	0.47 (0.22 - 1.00)	0.051	27	1.18 (0.65 - 2.14)	0.576
C/C	64	5	1.79 (0.59 - 5.39)	0.303	3	0.56 (0.16 - 1.94)	0.356	4	0.72 (0.24 - 2.17)	0.556
T/C & C/C	331				14	0.49 (0.24 - 0.98)	**0.045**	31	1.09 (0.62 - 1.94)	0.761
**NBS1_5UTR_(-352)_del(AGTA)**										
AGTA/AGTA	524	30	1	-	32	1	-	44	1	-
AGTA/-	58	5	1.47 (0.54 - 4.03)	0.457	0	0.00 (0.00 -)	0.997	7	1.61 (0.68 - 3.80)	0.283
-/-	2	0	0.00 (0.00 -)	0.999	0	0.00 (0.00 -)	0.999	0	0.00 (0.00 -)	0.999
AGTA/- & -/-	60	5	1.41 (0.52 - 3.86)	0.502	0	-	-	7	1.54 (0.65 - 3.63)	0.328
**NBS1_IVS3(+208)G/A**										
G/G	241	12	1	*0.400*	22	1	**-**	20	1	*0.752*
G/A	277	19	1.38 (0.65 - 2.95)	0.401	9	0.34 (0.15 - 0.76)	**0.008**	27	1.13 (0.61 - 2.08)	0.695
A/A	78	6	1.44 (0.51 - 4.05)	0.487	4	0.55 (0.18 - 1.68)	0.295	5	0.72 (0.26 - 2.00)	0.532
G/A & A/A	355				13	0.39 (0.19 - 0.79)	**0.009**			
**NBS1_3UTR(+273)G/A**										
G/G	268	11	1	*0.090*	19	1	-	22	1	-
G/A	263	21	1.99 (0.93 - 4.28)	0.077	15	0.76 (0.38 - 1.54)	0.450	27	1.22 (0.68 - 2.22)	0.505
A/A	63	5	2.04 (0.67 - 6.21)	0.212	2	0.48 (0.11 - 2.15)	0.339	2	0.39 (0.09 - 1.72)	0.215
G/A & A/A	326				17	0.71 (0.36 - 1.41)	0.329	29	1.07 (0.60 - 1.91)	0.832
**NBS1_X2_(102)_G/A**										
G/G	266	12	1	-	22	1	-	22	1	-
G/A	272	21	1.69 (0.81 - 3.55)	0.163	11	0.47 (0.22 - 0.99)	**0.047**	27	1.17 (0.64 - 2.11)	0.614
A/A	59	3	1.13 (0.30 - 4.23)	0.852	3	0.60 (0.17 - 2.10)	0.242	3	0.58 (0.17 - 2.01)	0.390
G/A & A/A	331	24	1.60 (0.78 - 3.29)	0.204	14	0.49 (0.24 - 0.98)	**0.045**		1.06 (0.60 - 1.89)	0.846
**NBS1_X5_(553)_G/C**										
G/G	255	12	1	-	21	1	-	22	1	-
G/C	270	20	1.58 (0.75 - 3.33)	0.233	12	0.52 (0.25 - 1.08)	0.078	28	1.18 (0.65 - 2.12)	0.590
C/C	57	3	1.10 (0.29 - 4.10)	0.888	2	0.41 (0.09 - 1.81)	0.239	3	0.57 (0.16 - 1.99)	0.378
G/C & C/C	327	23	1.49 (0.72 - 3.09)	0.281	14	0.50 (0.25 - 1.01)	0.052	31	1.07 (0.60 - 1.90)	0.824
**NBS1_X13_(2016)_A/G**										
A/A	247	12	1	-	20	1	-	21	1	-
A/G	265	20	1.56 (0.74 - 3.30)	0.242	11	0.49 (0.23 - 1.06)	0.070	26	1.12 (0.61 - 2.06)	0.708
G/G	55	3	1.11 (0.30 - 4.15)	0.879	2	0.42 (0.09 - 1.89)	0.259	3	0.59 (0.17 - 2.05)	0.402
A/G & G/G	320	23	1.49 (0.72 - 3.08)	0.288	13	0.48 (0.23 - 1.00)	**0.048**	29	1.03 (0.57 - 1.86)	0.931

SNP	Controls	All T-cell NHL			MF			PTCL		
	N	N	OR (95% CI)	p value	N	OR (95% CI)	p value	N	OR (95% CI)	p value
**NBS1_5(-905)T/C**										
T/T	266	20	1	*0.515*	13	1	-	7	1	-
T/C	267	24	1.24 (0.66 - 2.32)	0.499	12	0.95 (0.42 - 2.13)	0.894	10	1.46 (0.54 - 3.91)	0.454
C/C	64	6	1.25 (0.48 - 3.29)	0.647	3	0.97 (0.27 - 3.56)	0.967	2	1.18 (0.24 - 5.87)	0.840
T/C & C/C	331				15	0.95 (0.44 - 2.05)	0.899	12	1.40 (0.54 - 3.63)	0.486
**NBS1_5UTR_(-352)_del(AGTA)**										
AGTA/AGTA	524	44	1	-	24	1	-	18	1	-
AGTA/-	58	3	0.60 (0.18 - 2.02)	0.411	2	0.73 (0.17 - 3.23)	0.677	1	0.49 (0.06 - 3.79)	0.495
-/-	2	0	0.00 (0.00 -)	0.999	0	0.00 (0.00 -)	0.999	2	0.00 (0.00 -)	0.999
AGTA/- & -/-	60	3	-	-	2	-	-	3	-	-
**NBS1_IVS3(+208)G/A**										
G/G	241	18	1	*0.766*	11	1	-	7	1	-
G/A	277	25	1.22 (0.65 - 2.31)	0.540	14	1.11 (0.49 - 2.51)	0.805	10	1.24 (0.46 - 3.32)	0.673
A/A	78	6	1.03 (0.39 - 2.72)	0.948	3	0.83 (0.22 - 3.10)	0.786	1	0.43 (0.05 - 3.56)	0.433
G/A & A/A	355				17	1.05 (0.48 - 2.30)	0.907	11	1.06 (0.40 - 2.78)	0.911
**NBS1_3UTR(+273)G/A**										
G/G	268	21	1	-	14	1	-	7	1	-
G/A	263	26	1.41 (0.76 - 2.60)	0.272	13	1.06 (0.48 - 2.34)	0.884	10	1.55 (0.57 - 4.19)	0.387
A/A	63	2	0.38 (0.09 - 1.66)	0.197	1	0.28 (0.04 - 2.16)	0.221	1	0.58 (0.07 - 4.78)	0.609
G/A & A/A	326	28	1.18 (0.65 - 2.14)	0.597	14	0.88 (0.41 - 1.90)	0.742	11	1.34 (0.51 - 3.53)	0.557
**NBS1_X2_(102)_G/A**										
G/G	266	20	1	*0.582*	13	1	-	7	1	-
G/A	272	25	1.27 (0.68 - 2.35)	0.453	12	0.93 (0.41 - 2.08)	0.852	11	1.59 (0.60 - 4.17)	0.351
A/A	59	5	1.14 (0.41 - 3.19)	0.808	3	1.04 (0.28 - 3.82)	0.954	1	0.64 (0.08 - 5.38)	0.685
G/A & A/A	331				15	0.95 (0.44 - 2.04)	0.888	12	1.41 (0.55 - 3.66)	0.475
**NBS1_X5_(553)_G/C**										
G/G	255	20	1	*0.628*	13	1	-	7	1	-
G/C	270	25	1.24 (0.67 - 2.30)	0.498	12	0.91 (0.40 - 2.05)	0.818	11	1.54 (0.59 - 4.06)	0.381
C/C	57	5	1.11 (0.40 - 3.13)	0.839	3	1.02 (0.28 - 3.76)	0.975	1	0.63 (0.08 - 5.23)	0.665
G/C & C/C	327				15	0.93 (0.43 - 2.00)	0.852	12	1.38 (0.53 - 3.56)	0.512
**NBS1_X13_(2016)_A/G**										
A/A	247	20	1	-	13	1	-	7	1	-
A/G	265	24	1.16 (0.62 - 2.18)	0.635	12	0.90 (0.40 - 2.03)	0.792	10	1.35 (0.51 - 3.63)	0.547
G/G	55	4	0.88 (0.28 - 2.70)	0.816	2	0.66 (0.14 - 3.07)	0.598	1	0.62 (0.08 - 5.23)	0.664
A/G & G/G	320	28	1.12 (0.61 - 2.04)	0.732	14	0.85 (0.39 - 1.87)	0.691	11	1.23 (0.47 - 3.22)	0.681

OR = Odds Ratio, CI = Confidence Interval, NHL = Non-Hodgkin Lymphoma, DLBCL = Diffuse Large B-Cell Lymphoma, FL = Follicular Lymphoma, MZ/MALT = Marginal Zone lymphoma/Mucosa-Associated Lymphoma Tissue lymphoma,MCL = Mantle Cell lymphoma, SLL = Small Lymphocytic Lymphoma, LPL=Lymphoplasmacytic Lymphoma, Misc. B-cell = Miscellaneous B-cell lymphoma, MF = Mycosis Fungoides, PTCL = Peripheral T-Cell Lymphoma.

If less than 5 samples were in a category, the analysis is not valid and marked by "-". Analyses were not done for subtypes that had fewer than 5 heterozygotes and minor homozygotes combined. Analysis is adjusted for adjusted for gender, ethnicity, age, and residence.

p-value for test for trend is shown in italic type.

p-values less than 0.05 are in bold.

Additional File 9.3: Regression analysis for MRE11 SNPs in Caucasian samples only.

	Controls	All NHL			All B-cell NHL (with DLBC & FL)			DLBCL		
**SNP**	**N**	**N**	**OR (95% CI)**	**p value**	**N**	**OR (95% CI)**	**p value**	**N**	**OR (95% CI)**	**p value**
**MRE11_5(-1703)A/G**										
A/A	261	280	1	*0.963*	260	1	*0.972*	76	1	*0.431*
A/G	267	257	0.89 (0.70 - 1.13)	0.340	234	0.87 (0.681 - 1.12)	0.280	60	0.91 (0.63 - 1.32)	0.629
G/G	66	81	1.12 (0.78 - 1.62)	0.541	75	1.12 (0.77 - 1.63)	0.565	15	0.79 (0.42 - 1.47)	0.457
**MRE11_5(-1456)C/T**										
C/C	582	592	1	-	545	1	-	153	1	-
C/T	21	30	1.43 (0.80 - 2.53)	0.227	28	1.46 (0.82 - 2.62)	0.202	9	1.76 (0.78 - 3.96)	0.173
T/T	1	1	0.99 (0.06 - 15.98)	0.995	1	1.07 (0.07 - 17.30)	0.960	1	4.32 (0.26 - 72.48)	0.309
C/T & T/T	22	31	1.41 (0.80 - 2.47)	0.237	29	1.44 (0.82 - 2.56)	0.208	10	1.87 (0.86 - 4.08)	0.115
**MRE11_IVS2(+28)G/A**										
G/G	189	195	1	*0.980*	180	1	*0.906*	51	1	*0.751*
G/A	277	297	1.05 (0.81 - 1.36)	0.724	274	1.05 (0.80 - 1.36)	0.743	81	1.07 (0.72 - 1.59)	0.753
A/A	124	126	0.99 (0.71 - 1.36)	0.926	115	0.97 (0.70 - 1.35)	0.847	30	0.90 (0.54 - 1.49)	0.675
**MRE11_IVS9(-60)A/T**										
T/T	270	283	1	*0.963*	270	1	*0.958*	82	1	*0.419*
T/A	255	259	0.98 (0.77 - 1.25)	0.869	239	0.98 (0.77 - 1.26)	0.887	61	0.75 (0.51 - 1.09)	0.130
A/A	70	76	1.01 (0.70 - 1.46)	0.972	71	1.03 (0.71 - 1.50)	0.881	20	0.96 (0.55 - 1.68)	0.877

	Controls	FL			MZ/MALT			MCL		
**SNP**	**N**	**N**	**OR (95% CI)**	**p value**	**N**	**OR (95% CI)**	**p value**	**N**	**OR (95% CI)**	**p value**
**MRE11_5(-1703)A/G**										
A/A	261	81	1	*0.725*	25	1	*0.100*	17	1	*0.473*
A/G	267	69	0.84 (0.58 - 1.21)	0.341	29	1.20 (0.68 - 2.13)	0.527	20	1.18 (0.60 - 2.33)	0.629
G/G	66	22	1.03 (0.59 - 1.78)	0.919	13	1.95 (0.93 - 4.10)	0.076	6	1.400 (0.524 - 3.742)	0.502
**MRE11_5(-1456)C/T**										
C/C	582	165	1	-	67	-	-	41	-	-
C/T	21	9	1.38 (0.61 - 3.09)	0.441	1	-	-	2	-	-
T/T	1	0	0.00 (0.00 -)	0.100	0	-	-	0	-	-
C/T & T/T	22	9	1.34 (0.60 - 2.99)	0.481						
**MRE11_IVS2(+28)G/A**										
G/G	189	54	1	*0.967*	17	1	*0.144*	13	1	*0.849*
G/A	277	85	1.10 (0.75 - 1.63)	0.623	32	1.42 (0.75 - 2.66)	0.279	21	1.15 (0.55 - 2.36)	0.715
A/A	124	34	0.99 (0.61 - 1.61)	0.959	18	1.69 (0.83 - 3.46)	0.150	9	1.07 (0.44 - 2.60)	0.884
**MRE11_IVS9(-60)A/T**										
T/T	270	77	1	*0.866*	27	1	*0.625*	17	1	*0.366*
T/A	255	79	1.15 (0.80 - 1.66)	0.453	29	1.19 (0.68 - 2.11)	0.542	19	1.16 (0.58 - 2.31)	0.675
A/A	70	17	0.82 (0.45 - 1.47)	0.498	9	1.14 (0.51 - 2.58)	0.752	7	1.57 (0.62 - 4.00)	0.345

	Controls	SLL			LPL			Misc. B-cell		
**SNP**	**N**	**N**	**OR (95% CI)**	**p value**	**N**	**OR (95% CI)**	**p value**	**N**	**OR (95% CI)**	**p value**
**MRE11_5(-1703)A/G**										
A/A	261	17	1	*0.696*	21	1	-	23	1	-
A/G	267	14	0.85 (0.41 - 1.78)	0.670	11	0.51 (0.24 - 1.08)	**0.008**	21	0.93 (0.50 - 1.72)	0.811
G/G	66	6	1.45 (0.54 - 3.91)	0.459	4	0.77 (0.25 - 2.34)	0.644	4	1.61 (0.70 - 3.67)	0.262
A/G & G/G	333				15	0.56 (0.28 - 1.11)	0.097	25	1.06 (0.60 - 1.88)	0.838
**MRE11_5(-1456)C/T**										
C/C	582	33	-	-	36	-	-	50	-	-
C/T	21	4	-	-	0	-	-	3	-	-
T/T	1	0	-	-	0	-	-	0	-	-
**MRE11_IVS2(+28)G/A**										
G/G	189	14	1	*0.730*	17	1	**-**	14	1	*0.469*
G/A	277	15	0.78 (0.36 - 1.68)	0.527	14	0.57 (0.27 - 1.19)	0.131	26	1.34 (0.68 - 2.65)	0.398
A/A	124	8	0.89 (0.36 - 2.21)	0.803	4	0.35 (0.12 - 1.08)	0.068	12	1.32 (0.59 - 2.96)	0.502
G/A & A/A	401				18	0.50 (0.25 - 1.00)	**0.049**			
**MRE11_IVS9(-60)A/T**										
T/T	270	19	1	*0.703*	10	1	*0.051*	28	1	*0.506*
T/A	255	13	0.73 (0.34 - 1.53)	0.398	20	2.02 (0.92 - 4.44)	0.080	18	0.67 (0.36 - 1.24)	0.202
A/A	70	5	0.99 (0.35 - 2.80)	0.988	6	2.46 (0.86 - 7.09)	0.095	7	0.95 (0.40 - 2.28)	0.907

	Controls	All T-cell NHL			MF			PTCL		
**SNP**	**N**	**N**	**OR (95% CI)**	**p value**	**N**	**OR (95% CI)**	**p value**	**N**	**OR (95% CI)**	**p value**
**MRE11_5(-1703)A/G**										
A/A	261	20	1	*0.739*	12	1	-	6	1	-
A/G	267	23	1.13 (0.60 - 2.12)	0.708	12	0.98 (0.43 - 2.37)	0.958	11	1.83 (0.66 - 5.04)	0.245
G/G	66	6	1.12 (0.43 - 2.92)	0.824	4	1.23 (0.38 - 4.00)	0.726	1	0.64 (0.08 - 5.42)	0.681
A/G & G/G	333				16	1.03 (0.48 - 2.24)	0.938	12	1.58 (0.58 - 4.29)	0.369
**MRE11_5(-1456)C/T**										
C/C	582	47	-	-	27	-	-	17	-	-
C/T	21	2	-	-	1	-	-	1	-	-
T/T	1	0	-	-	0	-	-	0	-	-
**MRE11_IVS2(+28)G/A**										
G/G	189	15	1	*0.778*	10	1	*0.795*	5	1	-
G/A	277	23	1.04 (0.52 - 2.07)	0.908	12	0.81 (0.34 - 1.93)	0.633	9	1.22 (0.40 - 3.74)	0.724
A/A	124	11	1.13 (0.50 - 2.56)	0.773	6	0.90 (0.32 - 2.57)	0.845	4	1.27 (0.33 - 4.86)	0.724
G/A & A/A	401							13	1.24 (0.43 - 3.55)	0.691
**MRE11_IVS9(-60)A/T**										
T/T	270	23	1	*0.790*	11	1	-	10	1	-
T/A	255	20	1.03 (0.54 - 1.96)	0.930	13	1.48 (0.63 - 3.46)	0.367	6	0.64 (0.23 - 1.82)	0.402
A/A	70	5	0.82 (0.30 - 2.25)	0.695	4	1.40 (0.43 - 4.58)	0.579	1	0.36 (0.05 - 2.90)	0.339
T/A & A/A	325				17	1.46 (0.66 - 3.24)	0.355	7	0.58 (0.21 - 1.55)	0.275

OR = Odds Ratio, CI = Confidence Interval, NHL = Non-Hodgkin Lymphoma, DLBCL = Diffuse Large B-Cell Lymphoma, FL = Follicular Lymphoma, MZ/MALT = Marginal Zone lymphoma/Mucosa-Associated Lymphoma Tissue lymphoma,MCL = Mantle Cell lymphoma, SLL = Small Lymphocytic Lymphoma, LPL=Lymphoplasmacytic Lymphoma, Misc. B-cell = Miscellaneous B-cell lymphoma, MF = Mycosis Fungoides, PTCL = Peripheral T-Cell Lymphoma.

If less than 5 samples were in a category, the analysis is not valid and marked by "-". Analyses were not done for subtypes that had fewer than 5 heterozygotes and minor homozygotes combined. Analysis is adjusted for adjusted for gender, ethnicity, age, and residence.

p-value for test for trend is shown in italic type.

p-values less than 0.05 are in bold.

Additional File 9.4: Regression analysis for BLM SNPs in Caucasian samples only.

SNP	Controls N	All NHL N	All NHL OR (95% CI)	All NHL p value	All B-cell NHL (with DLBC and FL) N	All B-cell NHL (with DLBC and FL) OR (95% CI)	All B-cell NHL (with DLBC and FL) p value	DLBCL N	DLBCL OR (95% CI)	DLBCL p value
**BLM_IVS7(+388)C/T**										
C/C	517	534	1	-	494	1	-	140	1	-
C/T	78	83	1.03 (0.74 - 1.44)	0.857	75	1.01 (0.72 - 1.42)	0.968	22	1.04 (0.62 - 1.73)	0.891
T/T	3	1	0.33 (0.03 - 3.23)	0.341	1	0.36 (0.04 - 3.51)	0.379	0	0.00 (0.00 -)	0.999
C/T & T/T	81	84	1.01 (0.72 - 1.40)	0.973	76	0.98 (0.70 - 1.38)	0.923	22	1.01 (0.60 - 1.68)	0.978
**BLM_IVS7(+798)ins(T)**										
T/T	528	559	1	-	517	1	-	145	1	-
T/-	69	59	0.84 (0.58 - 1.22)	0.360	54	0.83 (0.57 - 1.22)	0.343	17	0.89 (0.51 - 1.57)	0.689
-/-	4	1	0.27 (0.03 - 2.42)	0.240	1	0.28 (0.03 - 2.53)	0.256	0	0.00 (0.00 -)	0.999
T/- & -/-	73	60	0.81 (0.56 - 1.17)	0.262	55	0.80 (0.55 - 1.17)	0.250	17	0.84 (0.48 - 1.47)	0.534
**BLM_IVS12(+7)T/C**										
T/T	316	325	1	*0.830*	301	1	*0.732*	88	1	*0.944*
T/C	243	244	0.97 (0.76 - 1.23)	0.785	224	0.96 (0.76 - 1.23)	0.758	58	0.85 (0.58 - 1.23)	0.389
C/C	45	46	0.98 (0.63 - 1.53)	0.940	41	0.95 (0.60 - 1.50)	0.816	15	1.23 (0.55 - 2.32)	0.533
**BLM_IVS21(-60)_del(GAA)**										
GAA/GAA	237	259	1	*0.540*	233	1	*0.630*	61	1	*0.968*
GAA/-	283	278	0.90 (0.71 - 1.15)	0.413	263	0.95 (0.74 - 1.22)	0.700	81	1.09 (0.75 - 1.60)	0.640
-/-	76	80	0.94 (0.65 - 1.35)	0.722	71	0.92 (0.64 - 1.34)	0.677	19	0.94 (0.53 - 1.69)	0.840

SNP	Controls N	FL N	FL OR (95% CI)	FL p value	MZ/MALT N	MZ/MALT OR (95% CI)	MZ/MALT p value	MCL N	MCL OR (95% CI)	MCL p value
**BLM_IVS7(+388)C/T**										
C/C	517	148	1	-	62	1	-	37	1	-
C/T	78	24	1.03 (0.63 - 1.70)	0.908	6	0.62 (0.26 - 1.49)	0.284	6	1.10 (0.45 - 2.74)	0.832
T/T	3	1	0.96 (0.10 - 9.65)	0.970	0	0.00 (0.00 -)	0.999	0	0.00 (0.00 -)	0.999
C/T & T/T	81	25	1.03 (0.623- 1.68)	0.915	6	0.59 (0.25 - 1.43)	0.244	6	1.07 (0.43 - 2.65)	0.885
**BLM_IVS7(+798)ins(T)**										
T/T	528	156	1	-	62	1	-	41	1	-
T/-	69	17	0.90 (0.51 - 1.58)	0.702	5	0.71 (0.27 - 1.84)	0.474	2	0.38 (0.09 - 1.62)	0.191
-/-	4	1	1.09 (0.12 - 10.39)	0.939	0	0.00 (0.00 -)	0.999	0	0.00 (0.00 -)	0.999
T/- & -/-	73	18	0.90 (0.52 - 1.58)	0.722	5	0.67 (0.26 - 1.75)	0.418	2	0.35 (0.08 - 1.51)	0.161
**BLM_IVS12(+7)T/C**										
T/T	316	88	1	*0.696*	33	1	*0.609*	18	1	*0.488*
T/C	243	68	0.99 (0.69 - 1.43)	0.975	29	1.18 (0.69 - 2.02)	0.546	22	1.62 (0.84 - 3.11)	0.152
C/C	45	16	1.21 (0.65 - 2.26)	0.554	5	1.12 (0.41 - 3.07)	0.823	5	0.78 (0.17 - 3.52)	0.746
**BLM_IVS21(-60)_del(GAA)**										
GAA/GAA	237	74	1	*0.727*	37	1	***0.026***	17	1	-
GAA/-	283	75	0.87 (0.60 - 1.26)	0.452	21	0.46(0.26 - 0.82)	**0.008**	24	1.24 (0.64 - 2.38)	0.525
-/-	76	24	0.98 (0.58 - 1.68)	0.949	8	0.56 (0.24 - 1.27)	0.162	2	0.33 (0.07 - 1.48)	0.149
GAA/- & -/-	359							26	1.03 (0.54 - 1.96)	0.935

SNP	Controls N	SLL N	SLL OR (95% CI)	SLL p value	LPL N	LPL OR (95% CI)	LPL p value	Misc. B-cell N	Misc. B-cell OR (95% CI)	Misc. B-cell p value
**BLM_IVS7(+388)C/T**										
C/C	517	28	1	-	31	1	-	48	1	-
C/T	78	7	1.76 (0.73 - 4.24)	0.209	5	1.15 (0.43 - 3.09)	0.778	5	0.73 (0.28 - 1.91)	0.524
T/T	3	0	0.00 (0.00 -)	0.999	0	0.00 (0.00 -)	0.999	0	0.00 (0.00 -)	0.999
C/T & T/T	81	7	1.71 (0.71 - 4.10)	0.234	5	1.10 (0.41 - 2.95)	0.845	5	0.71 (0.27 - 1.84)	0.476
**BLM_IVS7(+798)ins(T)**										
T/T	528	35	1	-	31	1	-	47	1	-
T/-	69	2	0.46 (0.11 - 1.97)	0.292	5	1.24 (0.46 - 3.35)	0.181	6	1.01 (0.41 - 2.48)	0.985
-/-	4	0	0.00 (0.00 -)	0.999	0	0.00 (0.00 -)	0.999	0	0.00 (0.00 -)	0.999
T/- & -/-	73	2	0.43 (0.10 - 1.87)	0.261	5	1.16 (0.43 - 3.13)	0.767	6	0.97 (0.39 - 2.37)	0.937
**BLM_IVS12(+7)T/C**										
T/T	316	19	1	-	19	1	-	36	1	**-**
T/C	243	15	1.03 (0.51 - 2.09)	0.942	16	1.15 (0.58 - 2.30)	0.694	16	0.58 (0.32 - 1.08)	0.086
C/C	45	2	0.75 (0.17 - 3.40)	0.713	1	0.00 (0.00 -)	0.998	1	0.20 (0.03 - 1.51)	0.119
T/C & C/C	288	17	0.98 (0.50 - 1.95)	0.964	17	0.96 (0.48 - 1.92)	0.916	17	0.52 (0.29 - 0.98)	**0.036**
**BLM_IVS21(-60)_del(GAA)**										
GAA/GAA	237	10	1	*0.101*	12	1	*0.601*	22	1	*0.490*
GAA/-	283	18	1.59 (0.71 - 3.55)	0.260	18	1.22 (0.60 - 2.73)	0.520	26	1.00 (0.55 - 1.81)	0.990
-/-	76	8	2.23 (0.83 - 6.00)	0.111	5	1.22 (0.41 - 3.64)	0.717	5	0.63 (0.23 - 1.75)	0.377

SNP	Controls	All T-cell NHL			MF			PTCL		
	N	N	OR (95% CI)	p value	N	OR (95% CI)	p value	N	OR (95% CI)	p value
**BLM_IVS7(+388)C/T**										
C/C	517	40	1	-	23	1	-	14	1	-
C/T	78	8	1.26 (0.56 - 2.82)	0.578	5	1.41 (0.51 - 3.87)	0.504	3	1.33 (0.37 - 4.78)	0.664
T/T	3	0	0.00 (0.00 -)	0.999	0	0.00 (0.00 -)	0.999	0	0.00 (0.00 -)	0.999
C/T & T/T	81	8	1.23 (0.55 - 2.74)	0.622	5	1.38 (0.50 - 3.78)	0.533		1.29 (0.36 - 4.62)	0.699
**BLM_IVS7(+798)ins(T)**										
**T/T**	528	42	1	-	22	1	-	18	-	
T/-	69	5	0.99 (0.38 - 2.62)	0.989	4	1.54 (0.51 - 4.66)	0.447	0	-	
-/-	4	0	0.00 (0.00 -)	0.999	0	0.00 (0.00 -)	0.999	0	-	
T/- & -/-	73	5	0.96 (0.36 - 2.53)	0.933	4	1.49 (0.49 - 4.51)	0.481			
**BLM_IVS12(+7)T/C**										
T/T	316	24	1	*0.572*	14	1	-	9	1	-
T/C	243	20	1.05 (0.56 - 1.95)	0.885	12	1.07 (0.49 - 2.38)	0.860	6	0.85 (0.30 - 2.43)	0.762
C/C	45	5	1.45 (0.52 - 4.05)	0.479	2	1.03 (0.22 - 4.72)	0.975	3	2.37 (0.61 - 9.21)	0.211
T/C & C/C	288				14	1.07 (0.50 - 2.29)	0.868	9	1.08 (0.42 - 2.77)	0.870
**BLM_IVS21(-60)_del(GAA)**										
GAA/GAA	237	26	1	*0.472*	15	1	*0.519*	8	1	-
GAA/-	283	15	0.49 (0.25 - 0.95)	0.034	8	0.46 (0.19 - 1.10)	0.081	7	0.73 (0.26 - 2.05)	0.547
-/-	76	9	1.05 (0.46 - 2.37)	0.908	5	1.03 (0.36 - 2.98)	0.955	4	1.41 (0.41 - 4.88)	0.587
GAA/- & -/-	359							11	0.88 (0.35 - 2.24)	0.793

OR = Odds Ratio, CI = Confidence Interval, NHL = Non-Hodgkin Lymphoma, DLBCL = Diffuse Large B-Cell Lymphoma, FL = Follicular Lymphoma, MZ/MALT = Marginal Zone lymphoma/Mucosa-Associated Lymphoma Tissue lymphoma,MCL = Mantle Cell lymphoma, SLL = Small Lymphocytic Lymphoma, LPL=Lymphoplasmacytic Lymphoma, Misc. B-cell = Miscellaneous B-cell lymphoma, MF = Mycosis Fungoides, PTCL = Peripheral T-Cell Lymphoma.

If less than 5 samples were in a category, the analysis is not valid and marked by "-". Analyses were not done for subtypes that had fewer than 5 heterozygotes and minor homozygotes combined. Analysis is adjusted for adjusted for gender, ethnicity, age, and residence.

p-value for test for trend is shown in italic type.

p-values less than 0.05 are in bold.
