# Supplementary material for: Genetic variation in the NBS1, MRE11, RAD50 and BLM genes and susceptibility to non-Hodgkin lymphoma
Source: BMC Med Genet. 2009 Nov 16;10:117. doi: 10.1186/1471-2350-10-117 (PMC2788526; doi:10.1186/1471-2350-10-117)
Supplement: Additional file 10 — Regression analysis for overall NHL in Asians and South-East Asian samples. Table containing statistical analysis results for overall NHL only. [file 1471-2350-10-117-S10.PDF]

Additional File 10: Regression analysis for overall NHL in Asians and South-East Asian samples.

	Controls	All NHL, Asian Samples			Controls	All NHL, SE-Asian Samples		
**SNP**	**N**	**N**	**OR (95% CI)**	**p value**	**N**	**N**	**OR (95% CI)**	**p value**
**RAD50_IVS4(+19)G/A**								
G/G	55	51	1	-	18	12	1	-
G/A	28	26	1.03 (0.52 - 2.01)	0.943	16	12	1.07 (0.35 - 3.27)	0.900
A/A	2	3	1.58 (0.25 - 10.01)	0.626	2	3	2.23 (0.29 - 17.27)	0.443
G/A & A/A	30	29	1.06 (0.55 - 2.05)	0.853	18	15	1.19 (0.41 - 3.48)	0.746
**RAD50_IVS7(-38)C/T**								
C/C	89	79	-	-	36	27	-	-
C/T	0	0	-	-	1	1	-	-
T/T	0	0	-	-	0	0	-	-
**RAD50_IVS22(+24)A/G**								
A/A	89	79	-	-	34	23	-	-
A/G	0	0	-	-	3	5	-	-
G/G	0	0	-	-	0	0	-	-
**RAD50_IVS22(+62)A/G**								
A/A	59	53	1	-	36	28	-	-
A/G	26	23	0.99 (0.49 - 1.98)	0.969	0	0	-	-
G/G	2	1	0.50 (0.04 - 5.88)	0.582	0	0	-	-
A/G & G/G	28	24	0.95 (0.48 - 1.87)	0.874				
**NBS1_5(-905)T/C**								
T/T	29	16	1	*0.172*	11	5	1	***0.020***
T/C	37	38	1.65 (0.76 - 3.61)	0.208	18	13	1.85 (0.47 - 7.27)	0.376
C/C	20	22	1.84 (0.77 - 4.43)	0.171	6	10	7.29 (1.39 - 38.11)	**0.019**
**NBS1_5UTR_(-352)_del(AGTA)**								
AGTA/AGTA	86	71	-	-	32	25	-	-
AGTA/-	0	0	-	-	0	0	-	-
-/-	0	0	-	-	0	0	-	-
**NBS1_IVS3(+208)G/A**								
G/G	15	9	1	*0.088*	10	6	1	*0.080*
G/A	39	28	1.14 (0.43 - 3.03)	0.798	17	11	1.15 (0.30 - 4.42)	0.837
A/A	32	40	1.97 (0.75 - 5.19)	0.169	9	12	3.54 (0.83 - 15.09)	0.087
**NBS1_3UTR(+273)G/A**								
G/G	31	20	1	***0.017***	18	6	1	-
G/A	47	39	1.17 (0.56 - 2.44)	0.674	13	16	6.85 (1.65 - 28.43)	**0.008**
A/A	6	19	4.39 (1.47 - 13.08)	**0.008**	4	5	12.68 (1.69 - 95.36)	**0.014**
G/A & A/A					17	21	7.58 (1.89 - 30.93)	**0.004**
**NBS1_X2_(102)_G/A**								
G/G	29	17	1	*0.109*	12	5	1	*0.048*
G/A	41	35	1.29 (0.60 - 2.80)	0.514	16	14	2.11 (0.54 - 8.18)	0.282
A/A	16	21	2.10 (0.86 - 5.15)	0.106	5	8	5.31 (1.02 - 27.58)	0.047
**NBS1_X5_(553)_G/C**								
G/G	28	18	1	*0.149*	11	5	1	*0.088*
G/C	42	35	1.14 (0.53 - 2.45)	0.739	17	13	1.59 (0.41 - 6.28)	0.505
C/C	15	20	1.99 (0.80 - 4.93)	0.137	6	8	4.20 (0.83 - 21.34)	0.083
**NBS1_X13_(2016)_A/G**								
A/A	26	16	1	*0.122*	10	6	1	*0.098*
A/G	38	32	1.22 (0.55 - 2.72)	0.628	15	13	1.26 (0.32 - 4.97)	0.740
G/G	12	18	2.23 (0.84 - 5.97)	0.109	4	8	4.59 (0.83 - 25.50)	0.082
**MRE11_5(-1703)A/G**								
A/A	33	32	1	*0.481*	16	12	1	-
A/G	44	35	0.79 (0.40 - 1.56)	0.493	14	12	1.37 (0.43 - 4.37)	0.594
G/G	11	8	0.75 (0.26 - 2.15)	0.594	5	3	0.71 (0.10 - 4.81)	0.723
A/G & G/G					19	15	1.21 (0.40 - 3.61)	0.737
**MRE11_5(-1456)C/T**								
C/C	89	78	-	-	37	29	-	-
C/T	0	0	-	-	0	0	-	-
T/T	0	0	-	-	0	0	-	-
**MRE11_IVS2(+28)G/A**								
G/G	21	22	1	*0.664*	10	11	1	*0.482*
G/A	49	35	0.60 (0.28 - 1.30)	0.196	14	9	0.49 (0.13 - 1.87)	0.293
A/A	15	15	0.98 (0.33 - 2.29)	0.785	12	8	0.66 (0.17 - 2.52)	0.541
**MRE11_IVS9(-60)A/T**								
T/T	49	40	1	*0.677*	23	14	1	-
T/A	33	29	1.08 (0.56 - 2.11)	0.818	10	12	1.5 (0.48 - 4.98)	0.465
A/A	5	6	1.31 (0.36 - 4.72)	0.680	1	1	1.69 (0.08 - 34.48)	0.733
T/A & A/A					11	13	1.56 (0.51 - 4.77)	0.434

SNP	Controls	All NHL, Asian Samples			Controls	All NHL, South-East-Asian Samples		
	N	N	OR (95% CI)	p value	N	N	OR (95% CI)	p value
**BLM_IVS7(+388)C/T**								
C/C	85	78	-	-	33	24	-	-
C/T	1	1	-	-	3	5	-	-
T/T	0	0	-	-	0	0	-	-
**BLM_IVS7(+798)ins(T)**								
T/T	85	78	-	-	36	26	-	-
T/-	0	1	-	-	0	1	-	-
-/-	0	0	-	-	0	0	-	-
**BLM_IVS12(+7)T/C**								
T/T	48	41	1	*0.456*	22	20	1	-
T/C	33	29	0.95 (0.49 - 1.86)	0.890	12	8	0.48 (0.14 - 1.61)	0.234
C/C	6	10	1.91 (0.61 - 5.91)	0.264	2	1	0.38 (0.03 - 5.24)	0.470
T/C & C/C					14	9	0.47 (0.15 - 1.48)	0.196
**BLM_IVS21(-60)_del(GAA)**								
GAA/GAA	67	54	1	-	8	6	1	-
GAA/-	20	22	1.45 (0.71 - 3.00)	0.311	23	14	0.80 (0.20 - 3.11)	0.743
-/-	1	2	2.41 (0.21 - 28.13)	0.482	4	7	1.87 (0.32 - 10.90)	0.485
GAA/- & -/-	21	24	1.50 (0.74 - 3.04)	0.257	27	21	0.97 (0.26 - 3.63)	0.965

If less than 5 samples were in a category, the analysis is not valid and marked by "-". Analyses were not done for subtypes that had fewer than 5 heterozygotes and minor homozygotes combined. Analysis is adjusted for adjusted for gender, ethnicity, age, and residence.
p-value for test for trend is shown in italic type.
p-values less than 0.05 are in bold.
